# SPANXB1 drives brain metastasis in breast cancer via MMP1 regulation: potential therapeutic insights with metformin

**DOI:** 10.1038/s41420-025-02721-4

**Published:** 2025-08-30

**Authors:** Qi Wang, Haofeng Wu, Zhaoyi Zhai, Dongliang Fang, Chun Yang, Li Liu, Xiaowei Jia, Baopu Du, Yingqi Lyu, Mingshan Zhang, Tao Lu, Lulu Wang, Yan Gao

**Affiliations:** 1 Department of Human Anatomy, School of Basic Medical Sciences, Capital Medical University, Beijing, China; 2 School of Basic Medical Sciences, Capital Medical University, Beijing, China; 3 Department of Experimental Center for Basic Medical Teaching, School of Basic Medical Sciences, Capital Medical University, Beijing, China; 4 Department of Neurosurgery, Sanbo Brain Hospital, Capital Medical University, Beijing, China; 5 Key Laboratory of Major Diseases in Children, Ministry of Education, Beijing, China

**Keywords:** Prognostic markers, Metastasis

## Abstract

Cancer-testicular antigens (CTAs) have been considered as potential prognostic biomarkers and therapeutic targets due to their specific expression and roles in tumorigenesis and metastasis. Among these, the function and mechanism of SPANXB1 in breast cancer brain metastasis (BCBM) remain poorly understood. In this study, we investigated the role of SPANXB1 in BCBM. Our results demonstrated that SPANXB1 was highly expressed in brain-tropic breast cancer cells and brain metastasis samples. Functional assays revealed that SPANXB1 promoted breast cancer cell invasion, migration, vasculogenic mimicry (VM) formation, and blood-brain barrier (BBB) extravasation, thereby accelerating the process of brain metastasis. Mechanistically, SPANXB1 facilitated chromatin opening at the MMP1 promoter region via histone H3R17me2 modification and upregulated transcription factor YY1, leading to increased MMP1 expression. In vivo experiments further confirmed the role of SPANXB1 in enhancing brain metastasis. Notably, metformin effectively inhibited the expression of SPANXB1 and MMP1, thereby attenuating BCBM progression. The present study indicates the potential of SPANXB1 as a diagnostic and therapeutic target for BCBM. Additionally, our findings suggest metformin as a promising therapeutic strategy for this highly aggressive disease.

## Introduction

Metastasis is the leading cause of mortality in cancer patients, and approximately half of breast cancer patients develop distant metastases [1]. The major metastatic sites of breast cancer include the lung, liver, brain, and bone, which exhibit specific characteristics leading to organ-specific tropism of cancer cells [2]. Brain metastasis is a major cause of mortality and is more frequent in patients with human epidermal growth factor receptor 2^+^ (HER2^+^) or triple-negative breast cancer (TNBC) [3–5]. However, patients with breast cancer and brain metastases remain disadvantaged by the paucity of clinical research in this area; in fact, such patients are often explicitly excluded from clinical trials [6]. Current therapeutic approaches for breast cancer brain metastasis (BCBM) include surgery, whole-brain radiation therapy, stereotactic radiosurgery, chemotherapy, and targeted therapeutics such as trastuzumab, an anti-HER2 antibody [7]. However, these treatments are invasive or are accompanied by high toxicity, and patients often respond poorly, with a low survival rate ranging from 2 to 16 months [7]. Therefore, there is an urgent need to develop effective treatments and identify reliable biomarkers [8–10].

Brain metastases often exhibit significant differences in gene expression compared to primary tumors [11, 12]. In our previous study, differentially expressed genes between MDA-MB-231 (231) cells and its brain metastatic variant MDA-MB-231BR (BR) cells were investigated, and SPANXB1 was found to be significantly upregulated in BR cells [13]. SPANX-B expresses a 108 amino acid protein and has been implicated in mammalian spermatogenesis [14]. Notably, SPANXB1 is a cancer-testis antigen (CTA), a class of tumor-associated antigens that is specifically expressed in a variety of human tumors and normal testes [14]. The high immunogenicity and tissue-specific expression of CTAs highlight their potential role in cancer detection and treatment [15]. Some CTAs are correlated with clinical stage or prognosis, highlighting their significance as oncogenes or biomarkers [16]. SPANXB1 is highly expressed in various malignant tumors, including breast cancer, lung cancer, melanoma, prostate cancer, bladder cancer, renal cell carcinoma, and testicular germ cell tumors [14, 15, 17, 18].

SPANXB1 has been shown to increase the migration and invasion of breast cancer cells and promote tumor metastasis [19, 20]. However, its specific functions and mechanisms in BCBM have not been fully explored. In the present study, we demonstrate that SPANXB1 upregulated the expression of the MMP1 gene through the transcription factor YY1 and the histone H3R17me2, which not only promoted the migration, invasion, and VM formation capabilities of BR cells but also increased blood-brain barrier (BBB) permeability by downregulating the expression of tight junction proteins in the BBB. Consequently, SPANXB1 facilitated the extravasation of breast cancer cells across the BBB and promoted the progression of brain metastasis. Our research contributes to the understanding of the molecular mechanisms underlying BCBM and suggests potential targets for future clinical treatment.

## Results

### SPANXB1 promotes breast cancer cell migration, invasion, and VM formation

**Fig. 1:**
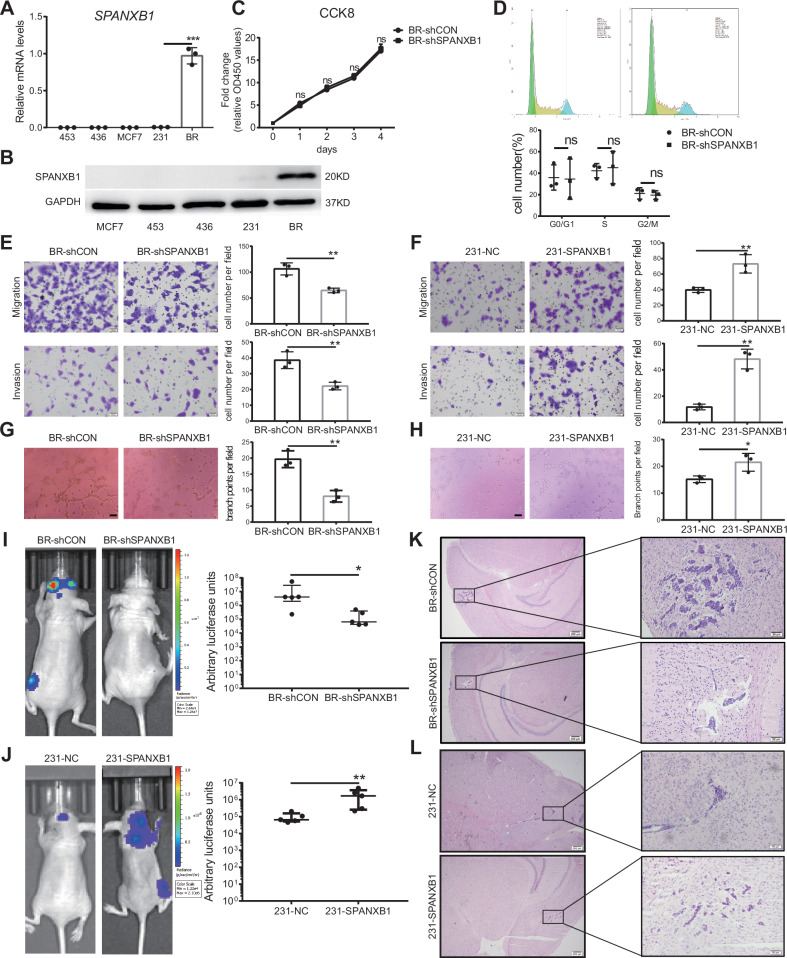
SPANXB1 is overexpressed in breast cancer cell lines and regulates cancer cell migration, invasion, VM formation and brain metastasis. **A**, **B** RT-qPCR and Western blot of SPANXB1 expression in breast cancer cell lines. **C** CCK-8 assay was used to detect cell proliferation in BR-shCON and BR-shSPANXB1 cells. *n* = 3 biological replicates. **D** Fluorescence-activated cell sorting analysis was performed to investigate the effects of apoptosis in BR-shCON (BR cells transfected with empty vector) and BR-shSPANXB1 cells, and the corresponding statistical plots are shown. *n* = 3 biological replicates. **E**, **F** Migration and invasion abilities of BR-shCON, BR-shSPANXB1, 231-NC, and 231-SPANXB1 cells detected by the trans-well assay. *n* = 3 biological replicates. **G**, **H** VM formation assay, BR-shCON, BR-shSPANXB1, 231-NC, and 231-SPANXB1 cells were seeded onto Matrigel and incubated for 5 h. VM structures were observed by phase-contrast microscopy. The number of branch points per field was quantified. Representative images and accompanying statistical plots are shown (scale bar, 100 µm). *n* = 3 biological replicates. **I**, **J** Luciferase-labeled BR-shCON, BR-shSPANXB1, 231-NC, and 231-SPANXB1 cells were injected into female nude mice via left ventricle injection. Brain metastasis was monitored by bioluminescence using IVIS after 4-week injection. *n* = 5/group. **K**, **L** HE staining of the brain from the indicated groups after harvest. *p < 0.05, **p < 0.01, ***p < 0.001. ns, not significance.

To investigate the key proteins and signaling pathways related to BCBM, we previously conducted RNA sequencing of 231 cells and the brain metastatic cell line BR cells [13]. Differential gene analysis has revealed that SPANXB1 expression is significantly higher in BR cells than in 231 cells [13]. Additionally, survival analysis indicated that high SPANXB1 expression is significantly associated with poor metastasis-free survival [13]. In the present study, we examined SPANXB1 expression in multiple breast cancer cell lines and found that SPANXB1 expression was low in MCF-7, 453, 436, and 231 cells but significantly increased in BR cells (Fig. 1A, B). These findings suggest that the expression level of SPANXB1 is increased in brain metastatic cells, indicating that it may be correlated with metastatic potential and poor prognosis.

To further explore the role of SPANXB1 in BCBM, we silenced SPANXB1 in BR cells and validated the knockdown efficiency at the mRNA level (Fig. S1A). We selected the most efficient sequence for stable knockdown via lentivirus and confirmed the effect at mRNA and protein levels (Fig. S1B, C). We also overexpressed SPANXB1 in 231 cells and confirmed the overexpression at mRNA and protein levels (Fig. S1D, E). First, we explored the effects of SPANXB1 on tumor cell-related biological functions in vitro. The results showed that knockdown of SPANXB1 did not affect the proliferation and apoptosis of BR cells but significantly inhibited their migration and invasion (Fig. 1C–E). Simultaneously, overexpression of SPANXB1 in 231 cells did not affect the proliferation but promoted cell migration and invasion (Figs. 1F and S1F). VM formation in tumors is associated with high tumor grade, tumor progression, and poor prognosis in patients with malignant tumors [21–23], and 231 cells could form VM in matrigel [24]. Here, we examined whether SPANXB1 affected the VM formation ability of cancer cells. SPANXB1 knockdown significantly inhibited VM formation of BR cells (Fig. 1G), while overexpression of SPANXB1 significantly enhanced VM formation of 231 cells (Fig. 1H).

Next, we conducted in vivo experiments to assess the effect of SPANXB1 on the brain metastasis of BR cells. Specifically, 4 weeks after injecting BR cells into the left ventricle of nude mice, we used bioluminescence imaging (BLI) technology to observe brain metastases. Knockdown of SPANXB1 reduced the formation of brain metastases of BR cells, whereas overexpression of SPANXB1 significantly increased the formation of brain metastases of 231 cells (Fig. 1I, J). These results were further supported by HE staining (Fig. 1K, L). In summary, these findings suggest that SPANXB1 may promote brain metastasis by facilitating breast cancer cell migration, invasion, and VM formation.

### SPANXB1 promotes breast cancer cell brain metastasis through MMP1

**Fig. 2:**
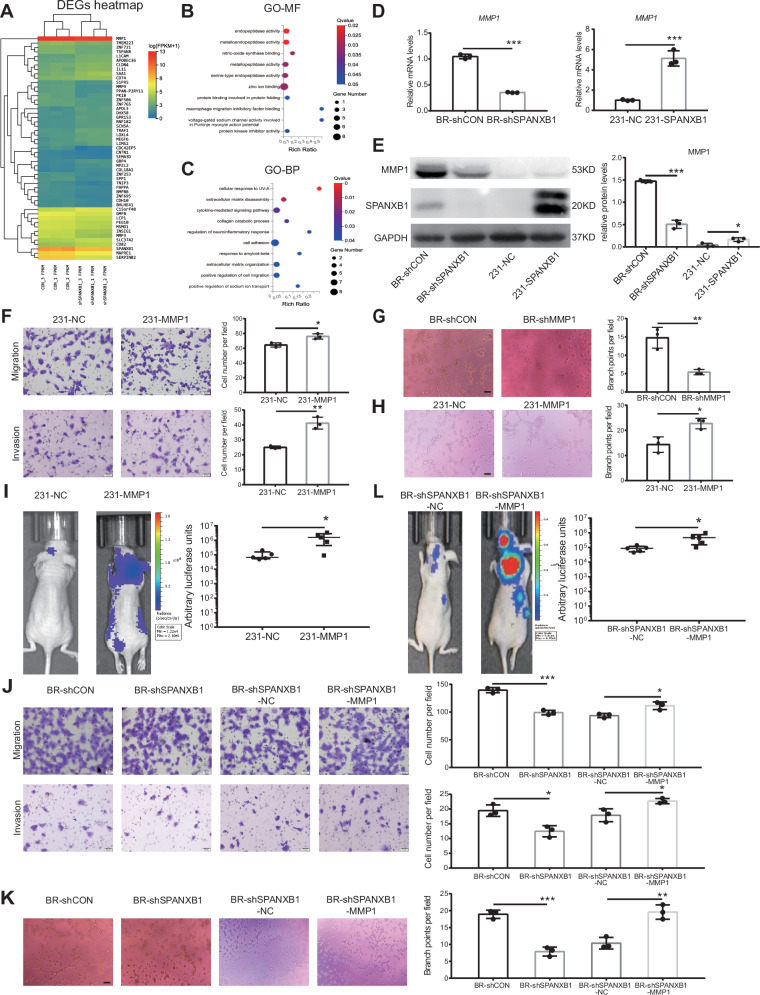
SPANXB1 promotes breast cancer cell migration, invasion, VM formation and brain metastasis through MMP1. **A** Heat map showing significantly differentially expressed genes in BR-shCON and BR-shSPANXB1 cells. **B** GO-MF enrichment analysis of differentially expressed genes between BR-shCON and BR-shSPANXB1 cells. **C** GO-BP enrichment analysis of differentially expressed genes between BR-shCON and BR-shSPANXB1 cells. **D**, **E** RT-qPCR and Western blot of MMP1 expression in BR-shCON, BR-shSPANXB1, 231-NC, and 231-SPANXB1 cells. **F** Migration and invasion abilities of 231-NC and 231-MMP1 cells detected by the trans-well assay. *n* = 3 biological replicates. **G**, **H** VM formation assay. BR-shCON, BR-shMMP1, 231-NC, 231-MMP1 cells were seeded onto Matrigel and incubated for 5 h. VM structures were observed by phase-contrast microscopy. The number of branch points per field was quantified. Representative images and accompanying statistical plots are shown (scale bar, 100 µm). *n* = 3 biological replicates. **I** Luciferase-labeled 231-NC and 231-MMP1 cells were injected into female nude mice via left ventricle injection. Brain metastasis was monitored by bioluminescence using IVIS after 4-week injection. *n* = 5/group. **J** Migration and invasion abilities of BR-shCON, BR-shSPANXB1, BR-shSPANXB1-NC, and BR-shSPANXB1-MMP1 cells detected by the trans-well assay. *n* = 3 biological replicates. **K** VM assay was used to detect VM formation in BR-shCON, BR-shSPANXB1, BR-shSPANXB1-NC, and BR-shSPANXB1-MMP1 cells. Representative images and accompanying statistical plots are shown (scale bar, 100 µm). *n* = 3 biological replicates. **L** Luciferase-labeled BR-shSPANXB1-NC and BR-shSPANXB1-MMP1 cells were injected into female nude mice via left ventricle injection. *n* = 5/group. Brain metastasis was monitored by bioluminescence using IVIS after 4-week injection. *p < 0.05, **p < 0.01, ***p < 0.001.

Previous studies have demonstrated that cancer cell migration, invasion, and VM formation are regulated by epithelial-mesenchymal transition (EMT) and matrix metalloproteinases (MMPs) [21, 25]. To investigate how SPANXB1 affects breast cancer cell brain metastasis, we performed RNA-seq to compare gene expression of SPANXB1-knockdown BR cells with control BR cells. The heatmap highlights significantly altered genes, notably the downregulation of MMP1 (Fig. 2A). GO enrichment analysis of these differentially expressed genes showed the significant enriched pathways, some of which were related to MMPs in molecular function (MF) or biological process (BP) (Fig. 2B, C). Previous sequencing revealed that the expression of MMP1 was also significantly higher in BR cells compared to 231 cells [13]. Western blot confirmed high levels of MMP1 and SPANXB1 in BR cells (Figs. 1B and S2C). To validate the effects of SPANXB1 on downstream gene expression, we knocked down SPANXB1 in BR cells and examined the expression of several classic genes associated with migration and invasion. The results demonstrated that the expression of MMP1 was significantly reduced at the mRNA and protein levels after SPANXB1 knockdown (Fig. S2A, B), while overexpression of SPANXB1 in 231 cells increased MMP1 at the mRNA and protein levels (Fig. 2D, E). To further validate this regulatory relationship, we performed SPANXB1 rescue experiments in BR-shSPANXB1 cells, which showed upregulated MMP1 expression at both mRNA and protein levels following SPANXB1 re-expression (Fig. S2D, E). Additionally, SPANXB1 overexpression in both 436 cells and 293T cells both led to increased MMP1 protein levels (Fig. S2F). These results suggested that SPANXB1 can regulate the expression of MMP1.

As a crucial effector molecule in tumor progression, MMP1 shows significant correlation with breast cancer aggressiveness when highly expressed, and exhibits the highest gene score during brain metastasis dissemination [26–30]. To investigate whether SPANXB1 promotes BCBM through MMP1, we validated the function of MMP1. We knocked down MMP1 in BR cells and overexpressed it in 231 cells, and the efficiency was verified at both mRNA and protein levels (Fig. S2G). MMP1 knockdown in BR cells and overexpression in 231 cells both showed no significant effect on cell proliferation (Fig. S2H). However, MMP1 knockdown significantly reduced the migration and invasion of BR cells (Fig. S2I) [29], while these capabilities of 231 cells were significantly enhanced after MMP1 overexpression (Fig. 2F). The VM formation ability of cancer cells were also regulated by MMP1. MMP1 knockdown in BR cells resulted in a significant reduction in VM formation, whereas MMP1 overexpression in 231 cells significantly increased this ability (Fig. 2G, H). We further investigated the role of MMP1 in breast cancer cell brain metastasis using in vivo experiments. Knockdown of MMP1 in BR cells reduced brain metastasis formation, while MMP1 overexpression in 231 cells significantly enhanced brain metastasis formation, as shown by BLI and HE staining (Figs. 2I and S2J-L). In summary, these findings suggest that MMP1 plays a crucial role in BCBM by promoting tumor cell migration, invasion, and VM formation.

To further explore whether the brain metastasis-promoting effect of SPANXB1 is mediated by MMP1, we conducted rescue experiments. We simultaneously knocked down SPANXB1 and overexpressed MMP1 in BR cells, confirming gene expression at the mRNA and protein levels (Fig. S2M). The results indicated that SPANXB1 knockdown reduced the migration, invasion, and VM formation capabilities of BR cells; however, these functions were restored upon further overexpression of MMP1 (Fig. 2J, K). We further conducted in vivo experiments, and the results of BLI showed that MMP1 overexpression restored the brain metastasis of BR cells after SPANXB1 knockdown (Fig. 2L). The results showed that SPANXB1 promotes BCBM by regulating MMP1 expression.

### SPANXB1 facilitates brain metastasis by enhancing the extravasation capacity of breast cancer cells across the BBB

**Fig. 3:**
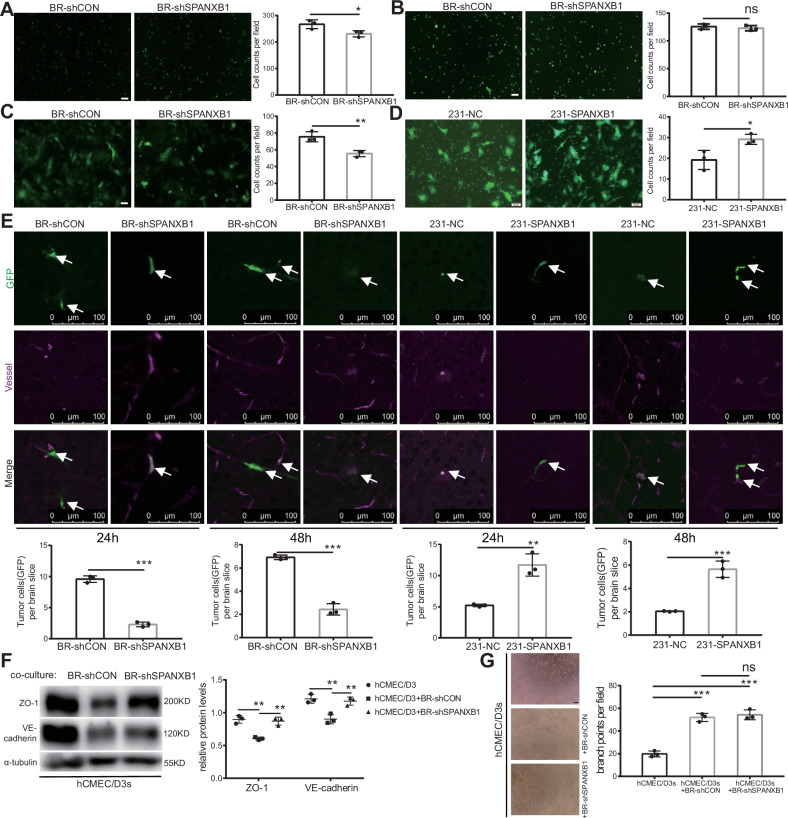
SPANXB1 facilitates cancer cell extravasation across the BBB. **A** Adhesion of BR-shCON and BR-shSPANXB1 cells (green) to hCMEC/D3s monolayers. Fluorescence images and statistical analyses are shown (scale bar, 100 µm). **B** Adhesion of BR-shCON and BR-shSPANXB1 cells (green) on the HUVECs monolayer. Fluorescence images and statistical analyses are shown (scale bar, 100 µm). *n* = 3 biological replicates. **C**, **D** Representative immunofluorescence images and quantitation of BR-shCON and BR-shSPANXB1 cells, 231-NC, and 231-SPANXB1 cells in trans-endothelial (hCMEC/D3s) migration assay (scale bar, 50 µm). *n* = 3 biological replicates. **E** 3D confocal images and statistical analysis of brain sections 24/48 h post-intracardiac injection. White arrows indicate cancer cells (green). *n* = 3 biological replicates. **F** Western blot of ZO-1 and VE-cadherin after co-culture of BR-shCON and BR-shSPANXB1 cells. **G** Tube formation assay, hCMEC/D3s cells were treated with conditioned medium generated from BR-shCON and BR-shSPANXB1 cells (24 h culture, followed by centrifugation) and seeded onto Matrigel. Tube formation was assessed after 4 h of incubation. The number of branch points per field was quantified. *n* = 3 biological replicates. *p < 0.05, **p < 0.01, ***p < 0.001. ns, not significance.

The ability of tumor cells to cross the BBB is crucial for BCBM development [31–34]. This process primarily occurs via the paracellular pathway through cerebrovascular endothelial cells [35, 36]. To investigate whether SPANXB1 influences the process of breast cancer cells crossing the BBB, we conducted adhesion and trans-endothelial migration assays. The adhesion experiments demonstrated that SPANXB1 knockdown significantly reduced the adhesion capacity of BR cells to hCMEC/D3s but had no effect on their adhesion to HUVECs (Fig. 3A, B). Trans-endothelial migration experiments revealed that knockdown of SPANXB1 significantly inhibited the ability of BR cells to migrate across hCMEC/D3s, whereas overexpression of SPANXB1 in 231 cells significantly enhanced their trans-endothelial migration capacity across hCMEC/D3s (Fig. 3C, D). We further used animal models to elucidate the impact of SPANXB1 on the ability of breast cancer cells to traverse the BBB. The results showed that BR cells successfully adhered to the endothelium and crossed the BBB 24 h post injection into the left ventricle. After SPANXB1 knockdown, the extravasation capacity of BR cells across the BBB was significantly reduced. Similar results were observed 48 h post injection into the left ventricle. Additionally, after SPANXB1 overexpression in 231 cells, the cells exhibited a significant increase in extravasation capacity of BR cells to cross the BBB (Fig. 3E). These findings suggest that SPANXB1 plays a pivotal role in breast cancer cell extravasation across the BBB.

Previous studies [37, 38] have indicated that tumor cells degrade the tight junctions of brain endothelial cells, weakening the endothelial barrier, and facilitating extravasation across the BBB to promote brain metastasis. In this study, we co-cultured BR cells with brain endothelial cells to examine the effect of SPANXB1 on tight junction proteins. Our results revealed a significant reduction of ZO-1 and VE-cadherin in brain endothelial cells co-cultured with BR cells, indicating a disruption of the endothelial barrier and enhanced cancer cell transmigration (Fig. 3F). Notably, SPANXB1 knockdown in BR cells partially restored these protein levels, suggesting that SPANXB1 plays a pivotal role in facilitating the disruption of tight junctions and endothelial barrier by BR cells (Fig. 3F). Concurrently, we investigated the impact of tumor cells on angiogenesis in cerebral microvascular endothelial cells using co-culture experiments. The results demonstrated that while BR cells significantly promoted angiogenesis in cerebral endothelial cells, SPANXB1 knockdown did not affect the angiogenic capacity of cerebral microvascular endothelial cells (Fig. 3G). This result indicates that SPANXB1 may not influence angiogenesis during brain metastasis. Overall, these findings suggest that SPANXB1 promotes the extravasation of breast cancer cells across the BBB by regulating the disruption of tight junctions in brain endothelial cells induced by tumor cells.

### SPANXB1 facilitates breast cancer cell extravasation across the BBB through MMP1

**Fig. 4:**
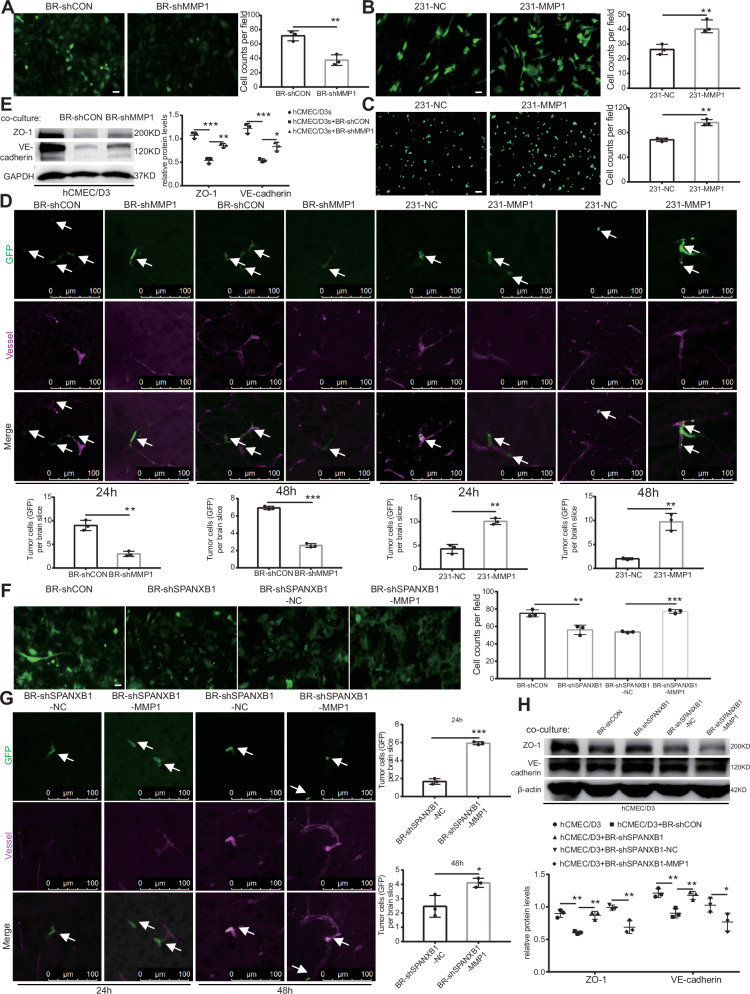
SPANXB1 facilitates cancer cell extravasation across the BBB through MMP1. **A**, **B** Immunofluorescence images and quantitation of BR-shCON and BR-shMMP1 cells, 231-NC, and 231-MMP1 cells in the trans-endothelial (hCMEC/D3s) migration assay. *n* = 3 biological replicates. **C** Qualitative adhesion of 231-NC and 231-MMP1 cells (green) on the hCMEC/D3s monolayer. Fluorescence images and statistical analyses are shown (scale bar, 100 µm). *n* = 3 biological replicates. **D** 3D confocal images and statistical analysis of cancer cells (green) in mouse brain on days 1 and 2 after intracardiac transplantation of BR-shCON, BR-shMMP1, 231-NC, and 231-MMP1 cells. White arrows indicate the cancer cells. *n* = 3 biological replicates. **E** Western blot of ZO-1 and VE-cadherin after the co-culture of BR-shCON and BR-shMMP1 cells. *n* = 3 biological replicates. **F** Immunofluorescence images and quantitation of BR-shCON, BR-shSPANXB1, BR-shSPANXB1-NC, and BR-shSPANXB1-MMP1 cells in trans-endothelial (hCMEC/D3s) migration assay. *n* = 3 biological replicates. **G** 3D confocal images and statistical analysis of cancer cells (green) in mouse brain on days 1 and 2 after intracardiac transplantation of BR-shSPANXB1-NC and BR-shSPANXB1-MMP1 cells. White arrows indicate the cancer cells. *n* = 3 biological replicates. **H** Western blot of ZO-1 and VE-cadherin after co-culture with BR-shCON, BR-shSPANXB1, BR-shSPANXB1-NC, and BR-shSPANXB1-MMP1 cells. *p < 0.05, **p < 0.01, ***p < 0.001.

We performed BBB extravasation experiments to determine the role of MMP1 in breast cancer cell extravasation. Trans-endothelial migration experiment demonstrated that knockdown of MMP1 in BR cells significantly inhibited their trans-endothelial migration capacity (Fig. 4A), while MMP1 overexpression in 231 cells enhanced their adhesion to brain endothelial cells and migration across brain endothelial cells (Fig. 4B, C). In vivo BBB extravasation experiments further demonstrated that BR cells successfully adhered to endothelial cells and crossed the BBB 24 h after left ventricular injection. However, MMP1 knockdown significantly reduced the ability of BR cells to cross the BBB (Fig. 4D). Additionally, MMP1 overexpression in 231 cells significantly enhanced BBB extravasation (Fig. 4D). The same results were observed after 48 h. These findings indicated that MMP1 plays a pivotal role in breast cancer cell extravasation across the BBB.

To further investigate whether the role of SPANXB1 in promoting breast cancer cell extravasation is mediated through MMP1, we conducted rescue experiments. In vitro extravasation experiments revealed that knockdown of SPANXB1 reduced the ability of BR cells to migrate across hCMEC/D3s, while overexpression of MMP1 significantly enhanced migration (Fig. 4F). In vivo experiments further demonstrated that BR cells with both SPANXB1 knockdown and MMP1 overexpression exhibited enhanced BBB extravasation 24 and 48 h post injection (Fig. 4G). These findings suggested that SPANXB1 promotes breast cancer cell extravasation across the BBB by regulating MMP1 expression.

In co-culture experiments with BR cells and brain endothelial cells, we also observed a significant decrease in ZO-1 and VE-cadherin expression in the brain microvascular endothelial cell layer, suggesting compromised endothelial barrier integrity. Knockdown of MMP1 in BR cells partially restored the levels of these tight junction proteins (Fig. 4E). Knockdown of SPANXB1 in BR cells partially restored ZO-1 and VE-cadherin levels in endothelial cells. However, simultaneous SPANXB1 knockdown and MMP1 overexpression increased the disruption of endothelial barrier integrity (Fig. 4H). These findings suggest that SPANXB1 may destroy the tight junctions of brain endothelial cells by regulating the expression of MMP1, thereby facilitating the extravasation of breast cancer cells across the BBB.

### SPANXB1 regulates the expression of MMP1 through the transcription factor YY1 and histone H3R17me2

**Fig. 5:**
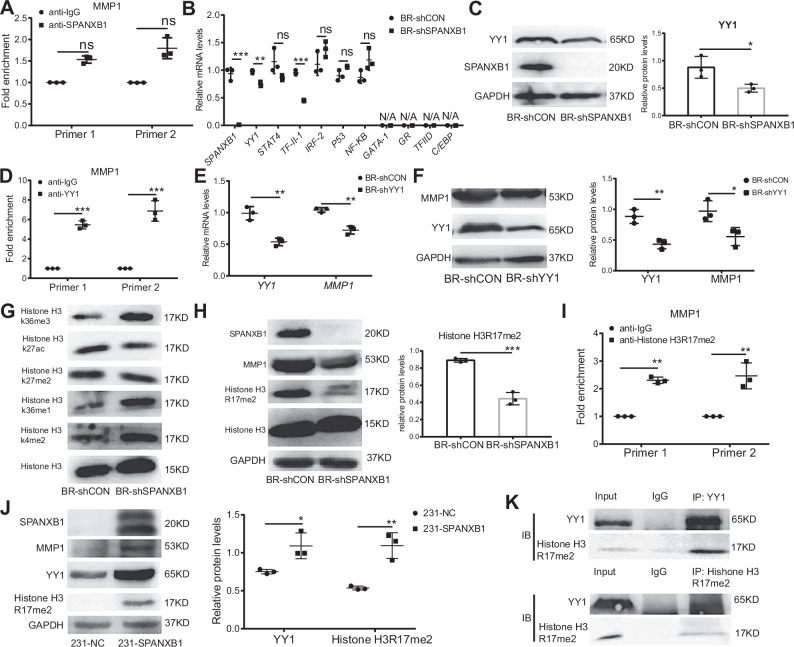
SPANXB1 regulates MMP1 expression through YY1 and Histone H3R17me2. **A** ChIP-qPCR was performed using an anti-SPANXB1 antibody in BR cells. **B** The mRNA levels of predicted transcription factors in BR-shCON and BR-shSPANXB1 cells detected by RT-qPCR. **C** The protein levels of YY1 in BR-shCON and BR-shSPANXB1 cells detected by Western blot. **D** ChIP-qPCR was performed using an anti-YY1 antibody in BR cells. **E** The mRNA levels of MMP1 in BR-shCON and BR-shYY1 cells detected by RT-qPCR. **F** The protein levels of MMP1 in BR-shCON and BR-shYY1 cells detected by Western blot. **G**, **H** Histone modification protein levels in BR-shCON and BR-shSPANXB1 cells detected by Western blot. **I** ChIP-qPCR was performed using an anti-histone H3R17me2 antibody in BR cells. **J** The protein levels of histone H3R17me2 in 231-NC and 231-SPANXB1 cells as detected by Western blot. **K** Evaluation of the interaction between YY1 and histone H3R17me2 by Co-IP. *p < 0.05, **p < 0.01, ***p < 0.001. ns, not significance.

We then investigated the mechanism by which SPANXB1 regulates MMP1 expression. Based on previous studies and our immunohistochemical results, SPANXB1 is a chromatin-associated protein that is primarily localized in the nucleus of BR cells (Fig. S3A) [14, 39]. SPANXB1 lacks the specific DNA-binding domains required for transcription factors, therefore, it cannot function as a transcription factor [40]. Furthermore, ChIP-qPCR experiments confirmed that SPANXB1 could not directly regulate the expression of MMP1 as a transcriptional co-activator (Fig. 5A). Next, we utilized transcription factor prediction websites to analyze upstream regulators of MMP1 and identified multiple candidate transcription factors, including GR, STAT4, TF-II, GAT4, and YY1 (Fig. S3B). Notably, SPANXB1 knockdown reduced YY1 expression in BR cells, as shown by qPCR, Western blot, and RNA-seq results (Figs. 5B, C and S3C). A previous study showed that YY1 regulates MMP1 in DLD-1 cells [41]. Therefore, we confirmed YY1 could bind to the MMP1 promoter region by ChIP-qPCR experiments and demonstrated that YY1 knockdown decreased MMP1 expression at both mRNA and protein levels (Fig. 5D–F). Thus, the results suggest that SPANXB1 indirectly influences MMP1 expression via YY1 in BR cells.

Considering the nuclear localization of SPANXB1 and the importance of histone modifications in gene regulation, we investigated the changes in classical histone modifications following SPANXB1 knockdown. We found that SPANXB1 knockdown in BR cells significantly decreased histone H3R17 di-methylation (Figs. 5G, H and S3D). Previous studies have shown that histone H3R17 di-methylation increases chromatin accessibility [37, 38, 42]. ChIP-qPCR results further confirmed that histone H3R17me2 could directly bind to the promoter region of MMP1 (Fig. 5I). Overexpression of SPANXB1 in 231 cells significantly increased both YY1 protein expression and histone H3R17me2 modification levels, suggesting that SPANXB1 regulates MMP1 expression via these two factors (Fig. 5J). Notably, overexpression of MMP1 in BR cells after SPANXB1 knockdown did not alter the levels of YY1 or histone H3R17me2, indicating that these are upstream factors of MMP1 (Fig. S3D). To further investigate the regulatory role of SPANXB1 on histone H3R17me2, we found that SPANXB1 knockdown led to downregulation of CARM1 (the specific methyltransferase for H3R17me2), which preliminarily demonstrated that SPANXB1 regulates histone H3R17me2 modification through modulating CARM1 expression (Fig. S3E). Co-IP experiments further verified an interaction between YY1 and histone H3R17me2 (Fig. 5K). Collectively, these results suggest that SPANXB1 regulates MMP1 expression by enhancing histone H3R17me2 modification and recruiting YY1 to the MMP1 promoter, thus facilitating MMP1 transcription.

### SPANXB1 regulates the PI3K-AKT signaling pathway via MMP1 and promotes breast cancer cell brain metastasis

To further explore the molecular mechanisms by which SPANXB1 influences BCBM, we performed KEGG enrichment analysis on genes significantly downregulated after SPANXB1 knockdown. This analysis revealed significant enrichment of the PI3K-AKT signaling pathway (Fig. S4A). Previous studies have shown that this pathway is associated with poor prognosis in patients with BCBM and formation of the brain microenvironment [8, 21]. The potential applications of PI3K inhibitors in breast cancer treatment have also been reported [43, 44]. We found that after SPANXB1 knockdown, AKT phosphorylation was significantly downregulated in BR cells (Fig. S4B). MMP1 knockdown in BR cells similarly decreased AKT phosphorylation (Fig. S4C). Conversely, overexpression of MMP1 or SPANXB1 in 231 cells resulted in marked upregulation of AKT phosphorylation (Fig. S4D). Through rescue experiments, we further confirmed that MMP1 overexpression effectively restored p-AKT levels (Fig. S4E). These findings suggested that SPANXB1 promotes the activation of PI3K-AKT signaling pathway by regulating the expression of MMP1, thereby facilitating BCBM.

### Metformin inhibits brain metastasis of BR cells by suppressing SPANXB1 and its downstream signaling pathways

**Fig. 6:**
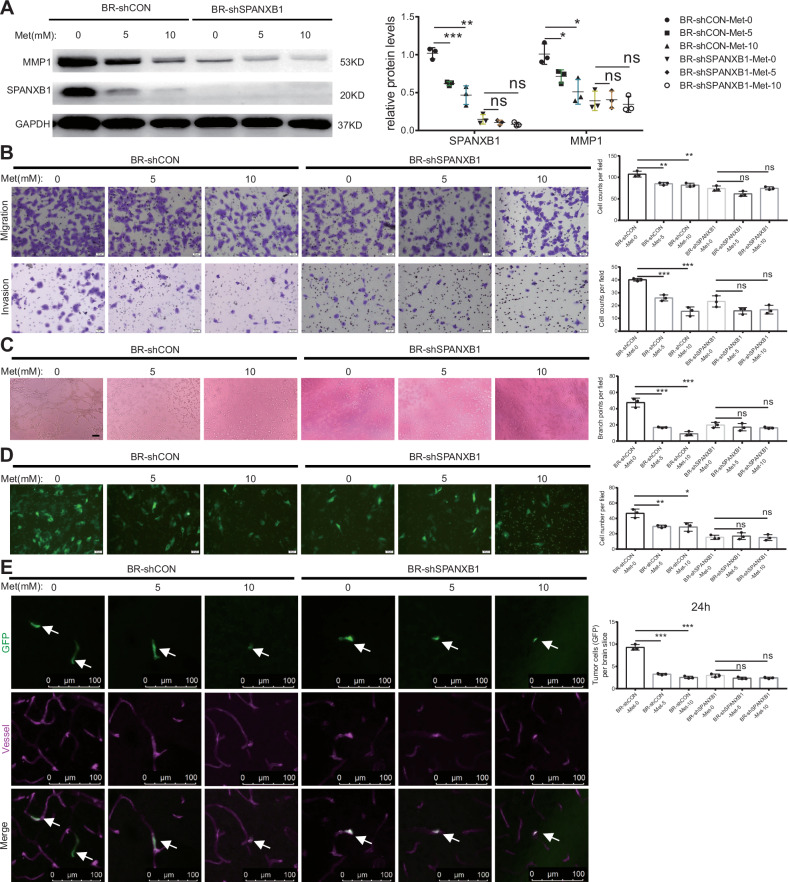
Effects of metformin on cell proliferation, migration, VM formation and BBB transmigration abilities of BR cells with and without SPANXB1 knockdown. **A** The protein levels of SPANXB1 and MMP1 in BR-shCON and BR-shSPANXB1 cells after metformin (0, 5, 10 mM, 48 h) treatment were determined using Western blot. **B** After metformin (0, 5, 10 mM, 48 h) treatment, cell migration and invasion were assessed using trans-well assays. Met: Metformin. *n* = 3 biological replicates. **C** 3D cell culture method was used to detect VM formation in BR-shCON and BR-shSPANXB1 cells treated with metformin (0, 5, 10 mM, 48 h). Representative images and accompanying statistical plots are shown (scale bar, 100 µm). *n* = 3 biological replicates. **D** Representative immunofluorescence images and quantitation of BR-shCON and BR-shSPANXB1 cells treated with metformin (0, 5, 10 mM, 48 h) in a trans-endothelial (hCMEC/D3s) migration assay (scale bar, 50 µm). *n* = 3 biological replicates. **E** BR-shCON and BR-shSPANXB1 cells were treated with metformin (0, 5, 10 mM, 48 h). 3D confocal images and statistical analysis of cancer cells (green) in the mouse brain 24 h post-injection. White arrows indicate the cancer cells. *n* = 3 biological replicates. *p < 0.05, **p < 0.01, ***p < 0.001. ns, not significance.

Metformin, commonly used for type 2 diabetes, has shown potential as a chemopreventive agent in cancer therapy [45–47]. It inhibits AKT/mTOR-mediated invasion [48, 49]. Our study demonstrates that metformin exerts dose-dependent inhibitory effects on both mRNA and protein expression of SPANXB1 in breast cancer cells (Figs. S5A and 6A). Intriguingly, while metformin treatment significantly suppressed MMP1 expression in control cells, this effect was abolished in SPANXB1-knockdown cells (Fig. 6A). Functional assays revealed that metformin treatment markedly attenuated migration, invasion, VM formation, trans-endothelial migration, and BBB extravasation (Fig. 6B–E). Notably, the antimetastatic efficacy of metformin was significantly compromised in SPANXB1-deficient cells, establishing SPANXB1 as a crucial mediator of metformin’s therapeutic effects (Fig. 6B–E). Consistently, rescue experiments in BR-shSPANXB1-MMP1 cells restored metformin’s inhibitory effects on migration and invasion (Fig. S5B), indicating that MMP1 reconstitution restored BR cell sensitivity to metformin. These results mechanistically validate SPANXB1 as an upstream transcriptional regulator of MMP1, functionally mediating metformin’s inhibitory effects on BR cell migration and invasion.

In conclusion, metformin significantly inhibits the brain metastasis potential of BR cells by suppressing the expression of SPANXB1, suggesting that SPANXB1 is a key molecular target for metformin on breast cancer cells.

### The expression of SPANXB1 and its downstream genes in primary and brain metastatic tumor samples of human breast cancer

**Fig. 7:**
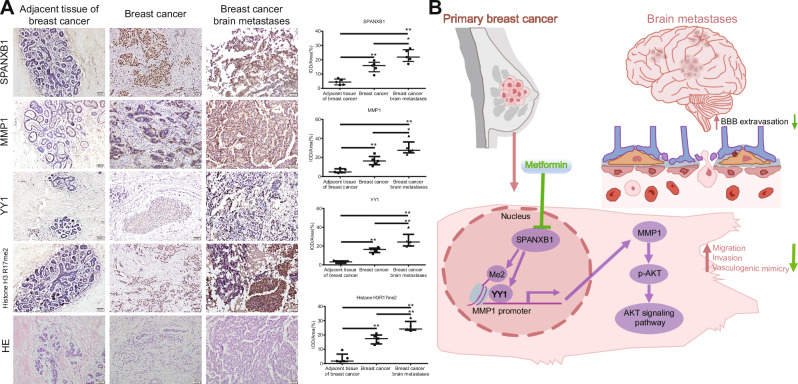
Expression of SPANXB1, MMP1, YY1 and Histone H3R17me2 in primary breast cancer and brain metastases. **A** HE staining results of adjacent non-cancerous tissues, primary breast cancer tissues, and brain metastatic tumor tissues, as well as IHC staining of SPANXB1, MMP1, YY1, and Histone H3R17me2 in these tissues, are presented (scale bar, 50 µm). *n* = 5/group. *p < 0.05, **p < 0.01. **B** Schematic diagram of our study.

To explore the expression of SPANXB1 in human BCBM, we first conducted HE staining on human adjacent non-cancerous tissues, primary breast cancer tissues and brain metastatic tumor samples. The results revealed abnormal cellular morphology, such as enlarged and pleomorphic nuclei in both primary and metastatic samples (Fig. 7A). IHC analysis showed increased Ki67 and CD31 positivity, indicating enhanced proliferation and angiogenesis in primary and metastatic tumors (Fig. S5B). Quantitative IHC analysis was used to measure SPANXB1, MMP1, YY1, and histone H3R17me2 expression. The expression levels were higher in primary tumors compared to non-cancerous tissues and even more elevated in brain metastases (Fig. 7A). These findings suggested that SPANXB1 and its downstream genes play a critical role in BCBM progression, thereby suggesting their potential as crucial clinical diagnostic markers and therapeutic targets.

In summary, this study demonstrated that SPANXB1 promotes MMP1 expression and subsequently induces migration, invasion, VM formation and BBB extravasation in breast cancer cells, thus promoting BCBM progression. Mechanistically, SPANXB1 mediates these effects through YY1 and histone H3R17me2 modification, which transcriptionally activates MMP1 and subsequently triggers PI3K-AKT pathway activation. Importantly, our findings revealed the therapeutic potential of metformin in suppressing BCBM by targeting the SPANXB1-MMP1 axis, providing new mechanistic insights into metformin’s anti-metastatic effects (Fig. 7B).

## Discussion

BCBM is a complex multi-step process involving EMT, intravasation, circulation survival, BBB extravasation, and brain colonization [27, 50, 51]. The present study investigated the role of SPANXB1, which is significantly upregulated in brain-seeking breast cancer cells, in the process of brain metastasis and its underlying molecular mechanism. We explored the effects of SPANXB1 on promoting breast cancer cell migration, invasion, VM, and BBB extravasation and further demonstrated that these effects were achieved through the upregulation of MMP1 expression. Our study provides the first demonstration that SPANXB1 drives BCBM through involving coordinated regulation of YY1 and histone H3R17me2 modification to activate MMP1 transcription. Importantly, we show that metformin can effectively inhibit BCBM progression by suppressing SPANXB1 expression. While other CTAs exhibit diverse oncogenic mechanisms across malignancies, such as SPAG9 driving prostate cancer progression via MAPK signaling, HSP70-2 promoting ovarian cancer growth and invasion, or CAGE enhancing autophagosome/spheroid formation in colon cancer through the CAGE-miR-140-5p-Wnt1 axis [16], SPANXB1 promotes BCBM through MMP1 regulation.

A key step in BCBM is BBB extravasation of circulating breast cancer cells [50]. The BBB comprises a monolayer of brain microvascular endothelial cells surrounded by basement membranes, pericytes, and astrocytes. These elements are tightly connected through tight and adherens junctions. Consequently, this structure has limited permeability [52, 53]. MMP1 is an enzyme capable of disrupting BBB integrity, and our study provides evidence that MMP1 can destroy tight junctions and increase BBB permeability [54]. Furthermore, we observed that SPANXB1 upregulates the expression of MMP1, which subsequently enhances the transmigration of breast cancer cells across the BBB and accelerates the progression of BCBM. These findings facilitate a deeper understanding of BCBM and suggest SPANXB1 as a promising therapeutic target for BCBM treatment.

YY1 is a multifunctional transcription factor that is upregulated in prostate cancer, colorectal cancer, and other tumors and is closely related to tumor progression [55, 56]. The histone H3R17me2 modification is an activating transcriptional signal [57, 58]. For the first time, we demonstrated that SPANXB1 can upregulate MMP1 by modulating YY1 and histone H3R17me2 modifications, thus providing a novel insight into the role of SPANXB1 as a chromatin-binding protein and its potential as a therapeutic target for managing tumor metastasis. We performed additional analyses to characterize histone H3K36me3 and H3K36me1 modifications following SPANXB1 knockdown (Fig. 5G). Both histone H3K36me1 and histone H3K36me3 showed exonic predominance and positive correlation with gene expression [59], with H3K36me3 serving as a characteristic marker of ongoing transcriptional elongation [60]. However, since these modifications are associated with transcriptional activation, which is inconsistent with our finding that SPANXB1 positively regulates MMP1, we primarily focused on the more pronounced reduction in histone H3R17me2, which directly correlated with MMP1 downregulation. This study significantly advances our understanding of SPANXB1’s regulatory effects on MMP1 expression.

Another significant finding of this study was that metformin markedly inhibited the expression of SPANXB1 and MMP1, thus suppressing BCBM. Metformin is the primary pharmacological agent used to treat type 2 diabetes [61, 62]. Metformin suppresses tumorigenesis by reducing serum glucose and insulin levels, acting on tumor cells and exerting its antitumor biological effects through AMPK-dependent and/or AMPK-independent signaling pathways [63]. Metformin has been shown to inhibit breast cancer cell proliferation, migration, invasion and progression, and improve chemosensitivity [46, 63, 64]. This study suggests that metformin suppresses migration, invasion, VM formation, trans-endothelial migration, and BBB extravasation by downregulating SPANXB1 and MMP1 expression. This finding not only provides new evidence regarding the anticancer mechanism of metformin but also implies its potential therapeutic application in BCBM. However, the supraphysiological concentrations (5–10 mM) required for anti-BCBM effects in this study far exceed the therapeutic doses for diabetes and may cause adverse effects such as gastrointestinal disturbances or lactic acidosis. Therefore, future studies should systematically evaluate metformin’s overall inhibitory effects on BCBM in animal models, investigate its clinical efficacy in BCBM patients and develop combination therapies with existing anticancer drugs to maintain therapeutic efficacy while reducing dosage requirements.

Our study systematically elucidates the critical role of the SPANXB1-MMP1 axis in BCBM and metformin’s potential for targeted intervention, but several limitations warrant further investigation. For clinical validation, while we confirmed elevated expression of SPANXB1 and its downstream effectors (YY1, H3R17me2, MMP1) in both primary breast tumors and brain metastases, the small sample size (*n* = 5 per group) necessitates validation in larger clinical cohorts. Future studies should integrate genomic and transcriptomic data from TCGA, METABRIC and other public databases with long-term follow-up information to comprehensively evaluate the correlation between SPANXB1 expression levels and BCBM prognosis.

The immunogenic properties of SPANXB1 make it a promising target for BCBM immunotherapy. SPANXB1 tumor-specific expression pattern and demonstrated ability to induce CD8^+^ T cell cytotoxic responses in melanoma and multiple myeloma provide theoretical foundation for its immunotherapeutic applications. However, key challenges in BCBM treatment include impaired MHC-I antigen presentation, immunosuppressive tumor microenvironment, and restricted immune cell infiltration due to the BBB. To fully exploit SPANXB1 therapeutic potential, combination strategies should be explored, such as: immune checkpoint inhibitors (e.g., anti-PD-1/CTLA-4), and optimized delivery systems (e.g., nanoparticle vaccines or localized T cell therapy).

Overall, this study, for the first time, revealed the molecular mechanism through which SPANXB1 promotes BCBM by upregulating MMP1, and has demonstrated the potential of metformin in inhibiting metastasis. These findings present new theoretical bases and molecular targets for early diagnosis, prognosis prediction, and targeted therapy of BCBM. Future research should further verify the feasibility of SPANXB1 as a therapeutic target, especially in clinical trials. By targeting the molecular mechanism of SPANXB1, this study offers a new therapeutic strategy for breast cancer patients with brain metastasis.

## Materials and methods

### Cell culture and reagents

HEK-293T cells, HUVECs and breast cancer cell lines MDA-MB-436 (436), MDA-MB-231 (231), MCF-7, MDA-MB-453 (453) were purchased from Hysigen Bioscience with STR profiles. hCMECD3s were purchased from ScienCell with STR profiles. All cell lines are free of mycoplasma contamination. The cell lines 231, 453, 436, MCF-7, and HEK-293T were cultured in Dulbecco’s modified Eagle’s medium (DMEM) (11965092, Gibco, USA) supplemented with 10% Fetal Bovine Serum (FBS) (A5670801, Gibco) and 1% penicillin-streptomycin (15140122, Gibco). MDA-MB-231BR (BR) cells, kindly provided by Dr. Patricia Steeg (National Cancer Institute, USA), were maintained in DMEM with 200 μg/mL G418 (Sigma-Aldrich), 10% FBS, and 1% penicillin-streptomycin. HUVECs were grown in RPMI-1640 medium (11875119, Gibco) supplemented with 10% FBS, and 1% penicillin-streptomycin. hCMEC/D3s [65] were cultured in an Endothelial Cell Medium supplemented with 5% FBS, 1% ECGF, and 1% penicillin-streptomycin. All cell culture conditions were at 37 °C, 5% CO_2_. Metformin (ALX-270-432, ENZO, China) was dissolved in deionized water and stored at −20 °C.

### Transfection of siRNA

BR cells were transfected with either negative control or SPANXB1-specific siRNA using Lipofectamine 2000 (11668019, Invitrogen, USA). Cells were harvested 48 h post transfection, and the siRNA target sequences are detailed in Table S1.

### Plasmid constructions

ShRNA sequences targeting SPANXB1, MMP1, and YY1 were cloned into the plvx-shRNA-puro vector (Likely Biotechnology, China). Overexpression constructs for SPANXB1 and MMP1 were inserted into the plvx-puro vector. Details of shRNA are in Table S2 and overexpression sequences referenced to NM_032461.4 (SPANXB1) and NM_002421.3 (MMP1).

### Lentivirus infection and cell transfection

Stable knockdown or overexpression of SPANXB1, MMP1, and YY1 was achieved in breast cancer cells (BR-shCON, BR-shSPANXB1, BR-shMMP1, BR-shYY1, 231-NC, 231-SPANXB1, 231-MMP1) using lentiviral vectors cloned and transfected into HEK-293T cells. Following infection, stable cell lines were selected with puromycin (ST551, Beyotime). Prior to adhesion and trans-endothelial migration assays, cells were infected with Rlv-ZsGreen-Puro-lentivirus (Likely Biotechnology, China). Cells for in vivo imaging were transfected with a luciferase-expressing vector (Shanghai Genechem, China). For routine transfections, cells seeded in 6-well plates were transfected using Lipofectamine 2000, with efficiency confirmed by qPCR and Western blot.

### Cell counting kit-8 (CCK-8) assay

The CCK-8 assay (C6005, NCM Biotech, China) was used to measure cell proliferation. Briefly, cells per well were seeded in 96-well plates, followed by the addition of 10 μL CCK-8 reagent to each well. After incubation at 37 °C for 2 h, absorbance was measured at 450 nm using a MULTISKAN MK3 microplate spectrophotometer (Thermo). Three independent biological replicates were performed using different batches of cells.

### Cell cycle and cell apoptosis analyses

BR-shCON and BR-shSPANXB1 cells were seeded in 6-well plates and harvested 12 h later. Cells were stained with Annexin V and propidium iodide (C1052, Beyotime) for 15 min in the dark and analyzed using CytExpert software. Three independent biological replicates were performed using different batches of cells to account for biological variability.

### Migration and invasion assays

Migration assays were performed using trans-wells with 8 μm pore polycarbonate membranes (Corning). For invasion assay, membranes were pre-coated with Matrigel (354248; Corning). Cells (3 × 10^4^ cells per well in 300 μL serum-free medium) were seeded in the upper chamber, while the lower chamber contained medium with 2% FBS (Migration) or 10% FBS (Invasion) as a chemoattractant. After 24 h incubation at 37 °C, cells were fixed and stained with 0.1% crystal violet, and counted. Three independent biological replicates were performed using different batches of cells.

### Tube formation assay and VM formation assay

Matrigel (354248; Corning, USA) was added to 96-well plates and solidified in a 37 °C, 5% CO2 incubator for 1 h. Conditioned medium was generated by culturing BR-shCON and BR-shSPANXB1 cells in complete medium for 24 h, followed by centrifugation to remove cell debris. hCMEC/D3 cells were treated with an equal volume of the conditioned medium and seeded onto the gel. After incubation for 4 h, the tube formation was assessed. For VM formation assay, cancer cells in complete medium were seeded onto the gel and incubated at 37 °C for 5 h. The formation of capillary-like structures was observed by phase-contrast microscopy (100× magnification). Three independent biological replicates were performed using different batches of cells.

### Adhesion assay

hCMEC/D3s or HUVECs were cultured in 12-well plates to form a monolayer over 3 days. Cancer cells (green) were then added and incubated at 37 °C for 20 min. After washing, cells were fixed with 4% paraformaldehyde for 10–15 min and examined under an Olympus inverted fluorescence microscope (Olympus) (100× magnification). The number of adherent cancer cells was counted. Three independent biological replicates were performed using different batches of cells.

### Trans-endothelial (hCMEC/D3s) migration assay

Cancer cell migration across hCMEC/D3s monolayers was assessed in vitro using 8.0 μm pore size trans-well inserts. hCMEC/D3s cells were seeded on the upper side of the inserts in 24-well plates and cultured for 48 h. GFP-expressing cancer cells were then added to the upper chamber in DMEM, with the lower chamber containing DMEM supplemented with 10% FBS. After 48 h at 37 °C in a 5% CO_2_ humidified atmosphere, cells were fixed with 4% paraformaldehyde for 10–15 min and were observed with an inverted fluorescence microscope (Olympus). Three independent biological replicates were performed using different batches of cells.

### In vitro co-culture model

hCMEC/D3s (5 × 10^5^) were first seeded in the lower chamber of the trans-well chamber for 48 h to form a monolayer. Breast cancer cells (5 × 10^4^) were then seeded in the upper chamber of the trans-well chamber. After 48 h of co-culture, only the endothelial cells from the lower chamber were collected for protein extraction and Western blot of ZO-1 and VE-cadherin expression. Three independent biological replicates were performed using different batches of cells.

### Animal model acquisition and group allocation

Female BALB/c nude mice aged 4–6 weeks were obtained from Beijing Vital River Laboratory Animal Technology Co., Ltd (Beijing, China) and housed under specific-pathogen-free conditions. Animals had adlibitum access to food and water, and were monitored daily for health status. All animal experiments were conducted in strict accordance with the guidelines established by the Animal Care and Use Committee of Capital medical university and adhered to the Guide for the Care and Use of Laboratory Animals. Mice were randomly divided into control and experimental groups. For animal studies, each experimental group contained 5 mice to achieve sufficient statistical power. In the MMP1 knockdown group, we used *n* = 3 mice per group to validate previously published findings.

### In vivo extravasation assay

Extravasation of GFP-expressing cancer cells into the brain parenchyma was examined using intracardiac transplantation combined with an ex vivo brain slice assay [66]. Cancer cells were injected into the circulation via intracardiac injection. At 24 and 48 h post injection, mice received Dylight594-labeled lectin (L32471, Invitrogen) to visualize blood vessels. Brains were fixed in 4% paraformaldehyde, cryopreserved in OCT at –80 °C after sinking in 30% sucrose, and then sectioned at 40 μm. Sections were covered with neutral resin, stored at 4 °C in the dark, and imaged with a Leica TCS SP8 confocal microscope. 3D images were reconstructed using Leica Application Suite X (LAS_X_4.7.0). Quantification of tumor cell extravasation was performed by calculating the average number of tumor cells that penetrate the BBB in each whole brain section. For representative images, typical microscopic fields showing characteristic BBB extravasation patterns were selected from each experimental group.

### Hematoxylin-eosin (HE) staining

Paraffin slides were deparaffinized, rehydrated, and stained with hematoxylin for 5 min, differentiated in 1% acid alcohol, and stained with eosin for 2 min. After dehydration and clearing, slides were mounted and imaged.

### Immunohistochemistry (IHC) analysis

Primary breast tumor samples (*n* = 5), adjacent normal tissue samples (*n* = 5), and brain metastases samples (*n* = 5) were fixed in 4% PFA, deparaffinized, rehydrated, and subjected to antigen retrieval. After quenching endogenous peroxidases with 3% H_2_O_2_ and blocking with 10% goat serum, slides were incubated overnight at 4 °C with primary antibodies (SPANXB1 (J926AA0022, Mibio, China), MMP1 (CY5330, Abways, China), Histone H3R17me2 (GTX55484, Genetex, China), Ki67 (PTM-5032, PTM Bio, China), CD31 (PTM-5336, PTM Bio), YY1 (CY5160, Abways)). They were then exposed to HRP-conjugated secondary antibodies (anti-rabbit (PV-9001, ZSGB-BIO, China), anti-mouse (PV-6002, ZSGB-BIO)) for 30 min at room temperature, developed with DAB (ZLI-9018, ZSGB-BIO), and counterstained with hematoxylin. The average optical density (AOD) from three high-quality fields per sample was analyzed.

### IVIS and analysis

BCBM was monitored using an IVIS Spectrum CT (PerkinElmer). Mice were intracardially injected with luciferase-expressing cancer cells in 0.1 mL PBS. 28 days post injection, 120 mg/kg D-luciferin (ST196, Beyotime) was administered intraperitoneally, and mice were anesthetized with isoflurane. Tumor bioluminescence was captured using the Spectrum CT bioluminescence channel and analyzed with Living Image® software (PerkinElmer) [67]. For in vivo imaging, mice were identified by ear tags, and the operator measuring luciferase signal intensity (using IVIS Imaging System) was unaware of the group allocation.

### RNA Sequencing and data analysis

RNA sequencing was performed as previously described [13], and the data were uploaded to GEO under accession number GSE281551. The mRNAs were identified as Differentially Expressed Genes if they met the following criteria: control FPKM value > 1, adjusted *P* value (*Q* value) <0.05 and |Log2 (fold-change)| >0.59. GO including biological processes (BP), molecular functions (MF) and KEGG enrichment analysis of annotated different expression gene were performed as previously described, the pathway with a criterion of adjusted *P* value (*Q* value) <0.05 was identified as significant [13].

### RNA extraction and RT-qPCR

Total RNA was extracted using TRIzol (15596018CN, Thermo Fisher) and reverse transcribed using RTv Reverse Transcriptase (RV101-01, Vazyme, China). Quantitative PCR (qPCR) was conducted using AceQ qPCR SYBR Green Master Mix (Q121-02, Vazyme) on a CFX Connect Real-Time PCR Detection System (Bio-Rad). GAPDH served as the reference gene, and relative expression changes were calculated using the 2^-∆∆Ct^ method. Primers are listed in Table S3. Three independent biological replicates were performed using different batches of cells.

### Western blot

Proteins were extracted, quantified using a Bicinchoninic Acid assay (P0011, Beyotime), and separated on 10% SDS-PAGE. Transferred to nitrocellulose membranes (Sigma-Aldrich; Merck KGaA), they were blocked with 5% milk and incubated overnight with primary antibodies. Primary antibodies targeting SPANXB1 (K108510P, solarbio, China), MMP9 (CY5205, Abways), MMP1 (CY5330, Abways), AKT (4691T, CST), p-AKT (D25E6, CST, USA), p-S6K (#9205, CST), YY1 (CY5160, Abways), Zona Occludens 1 (ZO-1) (21773-1-AP, Proteintech, China), VE-cadherin (CY3423, Abways), α-tubulin (YM3035, Immunoway, USA), Histone H3R17me2 (GTX55484, Genetex), Histone H3K36me3 (GTX54109, Genetex), Histone H3K27ac (GTX128944, Genetex), Histone H3K36me1 (GTX54107, Genetex), Histone H3K36me2 (GTX54108, Genetex), Histone H3K4me2 (GTX121915, Genetex), Histone H3 (K200033M, solarbio), GAPDH (60004-1-IG, Proteintech), β-actin (66009-1-IG, Proteintech). After washing, membranes were incubated with HRP-conjugated secondary antibodies (anti-mouse (ab6728, Abcam, UK) and anti-rabbit (ab6721, Abcam)) and visualized using a chemiluminescence system (Fusion Solo 5, VILBER LOURMAT). Protein quantification was normalized by calculating the ratio of target protein band intensity to internal reference protein band intensity using ImageJ software to correct for technical variations. Three independent biological replicates were performed using different batches of cells.

### Co-immunoprecipitation (Co-IP)

Cells were lysed, and supernatants incubated overnight at 4 °C with specific antibodies or IgG (#2729, CST). Protein A/G Magnetic Beads (HY-K0202, MCE, USA) were added, and complexes pulled down and analyzed by Western blot. Three independent biological replicates were performed using different batches of cells.

### Chromatin immunoprecipitation quantitative PCR (ChIP-qPCR) assay

Cells fixed in 1% formaldehyde were lysed, sonicated, and incubated with antibodies or IgG. Protein-DNA complexes were pulled down with Protein A/G beads, reverse cross-linked, and DNA purified for qPCR analysis using primers targeting the MMP1 promoter. The primer sequences used to detect the binding sites along the MMP1 promoter are listed in Table S4. The fold enrichment was calculated using the 2^ΔΔCq method (ΔΔCq = Cq[IgG] - Cq[antibody group]), with a fold enrichment >2 considered statistically significant. Three independent biological replicates were performed using different batches of cells.

### Metformin for the detection of BR cell biological function, and BBB extravasation

BR-shCON, BR-shSPANXB1, BR-shSPANXB1-NC and BR-shSPANXB1-MMP1 cells were treated with 5 mM and 10 mM Metformin for 48 h [68]. Post-treatment, cells underwent assays for proliferation, migration, invasion, VM formation, trans-endothelial migration, and BBB extravasation. Protein levels were assessed by Western blot. Three independent biological replicates were performed using different batches of cells.

### Statistical analysis

Three independent biological replicates were performed using different batches of cells to account for biological variability. Comparisons among multiple groups were analyzed by one-way analysis of variance (ANOVA). Statistical analysis was performed using unpaired, two-tailed Student’s *t* test or Mann–Whitney’s U test, as appropriate using the GraphPad Prism 7.0. Data are presented as mean ± SD or median ± interquartile (IQR). A *p*-value less than 0.05 was considered statistically significant.

## Supplementary information

supplementary legends

Figure s1

Figure s2

Figure s3

Figure s4

Figure s5

Table S1-4

Original Western Blots

## Data Availability

RNA-seq data generated in this study are publicly available in the GEO dataset under accession number GSE281551.
