# Supplementary material for: SPANXB1 drives brain metastasis in breast cancer via MMP1 regulation: potential therapeutic insights with metformin
Source: Cell Death Discov. 2025 Aug 30;11:418. doi: 10.1038/s41420-025-02721-4 (PMC12398519; doi:10.1038/s41420-025-02721-4)
Supplement: Supplementary file 1 — supplementary legends [file 41420_2025_2721_MOESM1_ESM.doc]

**Supplementary Figure 1. Verification of SPANXB1 expression and knockdown/overexpression efficiency in BR and 231 cells. A** RT-qPCR analysis of SPANXB1 expression after SPANXB1 silencing in BR cells. **B, C** RT-qPCR and Western blot of SPANXB1 expression after SPANXB1 knockdown in BR cells. **D, E** RT-qPCR and Western blot of SPANXB1 expression after SPANXB1 overexpression in 231 cells. **F** CCK-8 assay was used to detect cell proliferation in BR-shCON and BR-shSPANXB1 cells. n = 3 biological replicates. ***p < 0.001. ns, not significance.

**Supplementary Figure 2. MMP1 promotes BR migration, invasion, and brain metastasis.**

**A, B** RT-qPCR and Western blot of migration-related gene expression in BR-shCON and BR-shSPANXB1 cells. **C** Western blot of MMP1 expression in breast cancer cell lines. **D, E** RT-qPCR and Western blot of SPANXB1 and MMP1 expression in BR-shSPANXB1-NC and BR-shSPANXB1-MMP1 cells. **F** Western blot of SPANXB1 and MMP1 expression in 436-NC, 436-SPANXB1, 293T-NC, and 293T-SPANXB1 cells. **G** RT-qPCR and Western blot of MMP1 expression after MMP1 knockdown in BR cells and overexpression in 231 cells. **H** CCK-8 assay was used to detect cell proliferation in BR-shCON, BR-shMMP1, 231-NC, 231-MMP1 cells. n = 3 biological replicates. **I** Migration and invasion abilities of BR-shCON and BR-shMMP1 cells detected by the trans-well assay. n = 3 biological replicates. **J** Luciferase-labeled BR-shCON and BR-shMMP1 cells were injected into female nude mice via left ventricle injection. Brain metastasis was monitored by bioluminescence using IVIS after 4-week injection. n = 3/group. **K, L** HE staining of brain metastases from BR-shCON and BR-shMMP1 cells, 231-NC and 231-MMP1 cells upon harvest. *p < 0.05, **p < 0.01, ***p < 0.001. ns, not significance.

**Supplementary Figure 3. Mechanism study of the regulation of MMP1 by SPANXB1.**

**A** Representative images of ICC staining showing the expression of SPANXB1 in BR-shCON and BR-shSPANXB1 cells. **B** Prediction of transcription factors upstream of MMP1 (https://alggen.lsi.upc.es/). **C** Expression of YY1 in BR cells after SPANXB1 knockdown as determined by RNA-seq analysis (GSE281551). **D** Western blot of MMP1, YY1, and Histone H3R17me2 expression in BR-shCON, BR-shSPANXB1, BR-shSPANXB1-NC, and BR-shSPANXB1-MMP1 cells. E Western blot of CARM1 expression in BR-shCON, BR-shSPANXB1. **p < 0.01. ns, not significance.

**Supplementary Figure 4. KEGG pathway analysis and protein expression validation after SPANXB1 knockdown.**

**A** KEGG enrichment analysis of the differentially downregulated genes in BR-shCON and BR-shSPANXB1 cells. **B** Western blot of PI3K-AKT signal pathway proteins in BR-shCON and BR-shSPANXB1 cells. **C** Western blot of PI3K-AKT signal pathway protein levels in BR-shCON and BR-shMMP1 cells. **D** Western blot of PI3K-AKT signal pathway protein levels in 231-NC, 231-MMP1, and 231-SPANXB1 cells. **E** Western blot of PI3K-AKT signal pathway protein levels in BR-shSPANXB1-NC and BR-shSPANXB1-MMP1 cells. *p < 0.05, **p < 0.01. ns, not significance.

**Supplementary Figure 5. Expression of Ki67 and CD31 in primary breast cancer and brain metastases.**

**A** The mRNA level of SPANXB1 in BR-shCON and BR-shSPANXB1 cells treated with metformin detected by RT-qPCR. **B** BR-shSPANXB1-NC and BR-shSPANXB1-MMP1 cells were treated with metformin (0, 5, 10mM) for 48h. Cell migration and invasion were assessed using a trans-well assay. Met: Metformin. n = 3 biological replicates. **C** Representative images of IHC staining showing the expression of Ki67 and CD31 in primary breast cancer, brain metastases, and adjacent breast cancer tissues. n = 5/group. **p < 0.01, ***p < 0.001. ns, not significance.

**Supplementary Table 1**. **Sequences of siRNA targeting SPANXB1.**

**Supplementary Table 2. Sequences of shRNA for knockdown.**

**Supplementary Table 3. Primer sequences for qPCR analysis.**

**Supplementary Table 4. Primer sequences for ChIP-qPCR targeting MMP1 gene.**
