# Supplementary figures and images for: SPANXB1 drives brain metastasis in breast cancer via MMP1 regulation: potential therapeutic insights with metformin
Source: Cell Death Discov. 2025 Aug 30;11:418. doi: 10.1038/s41420-025-02721-4 (PMC12398519; doi:10.1038/s41420-025-02721-4)

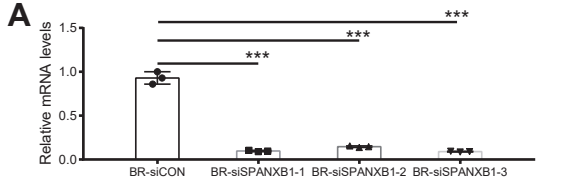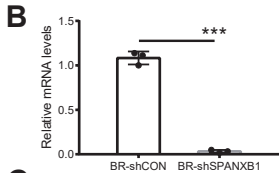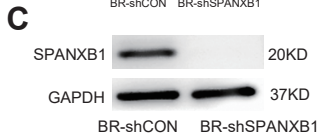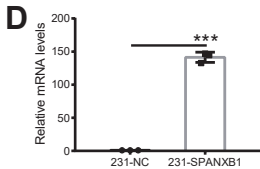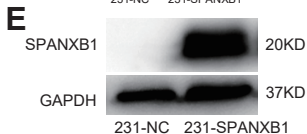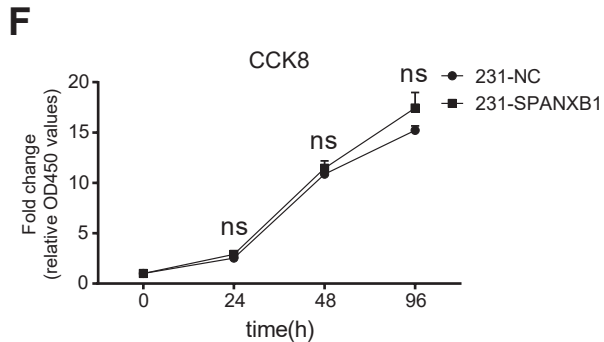

Supplement: Supplementary file 2 — Figure s1 [file 41420_2025_2721_MOESM2_ESM.pdf]

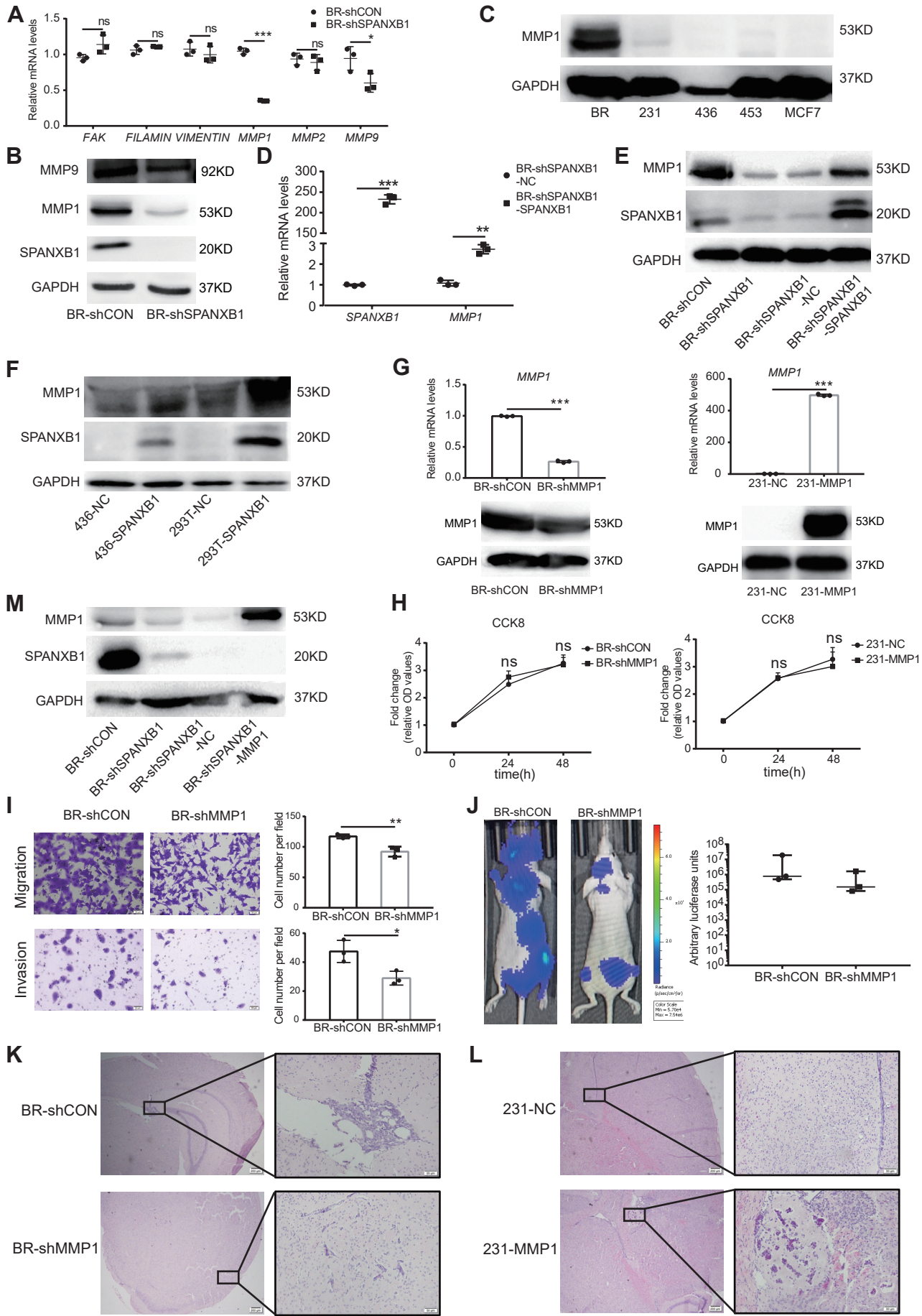

Supplement: Supplementary file 3 — Figure s2 [file 41420_2025_2721_MOESM3_ESM.pdf]

**A**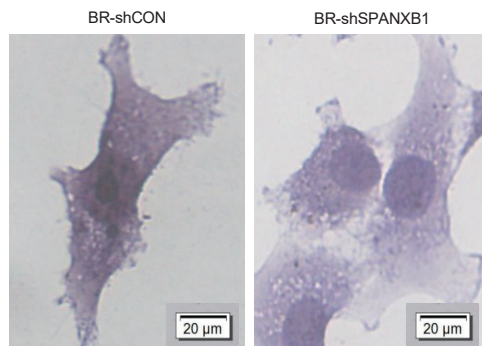**D**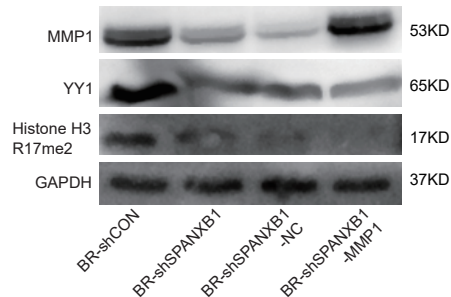**B**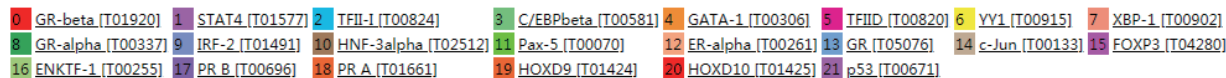**C**

| Gene ID | Gene Symbol | Type | log2 (shSPANXB1 / CON) | Qvalue (shSPANXB1 / CON) |
|---------|-------------|------|------------------------|--------------------------|
| 7528    | 'YY1'       | mRNA | -0.215474577           | 3.31E-04                 |

**E**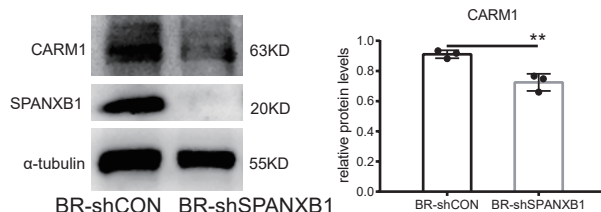

Supplement: Supplementary file 4 — Figure s3 [file 41420_2025_2721_MOESM4_ESM.pdf]

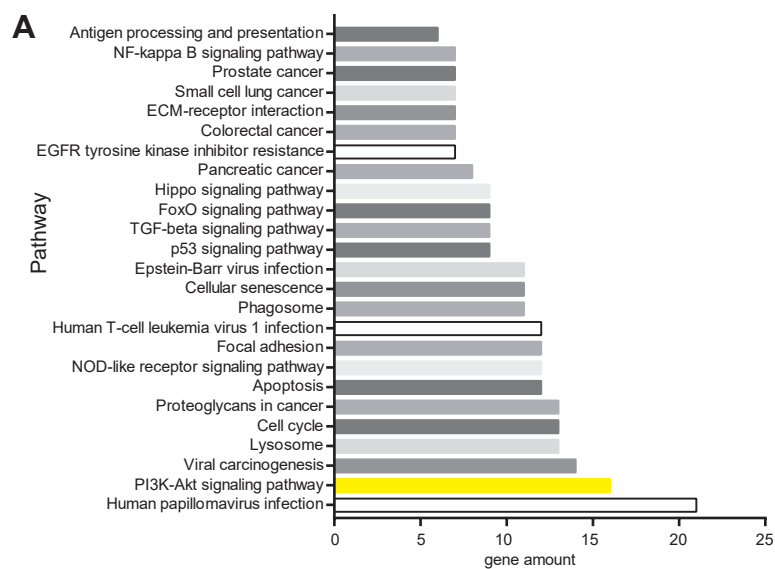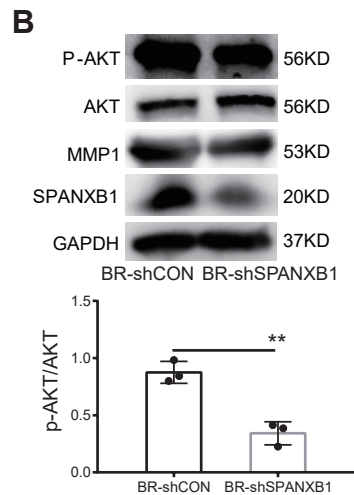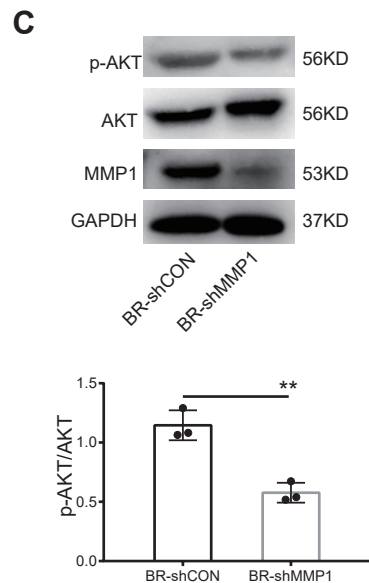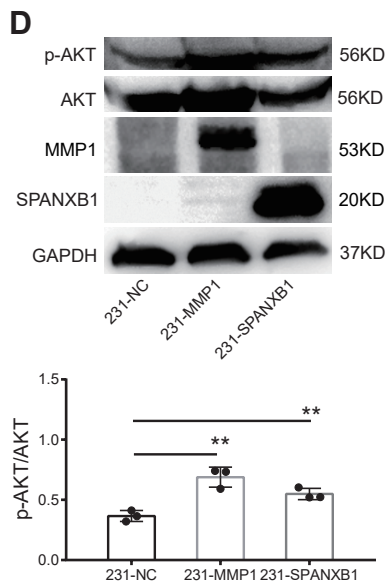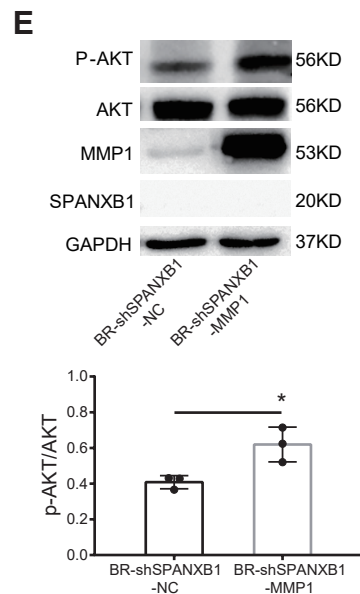

Supplement: Supplementary file 5 — Figure s4 [file 41420_2025_2721_MOESM5_ESM.pdf]

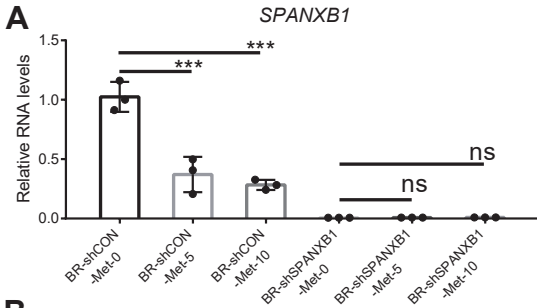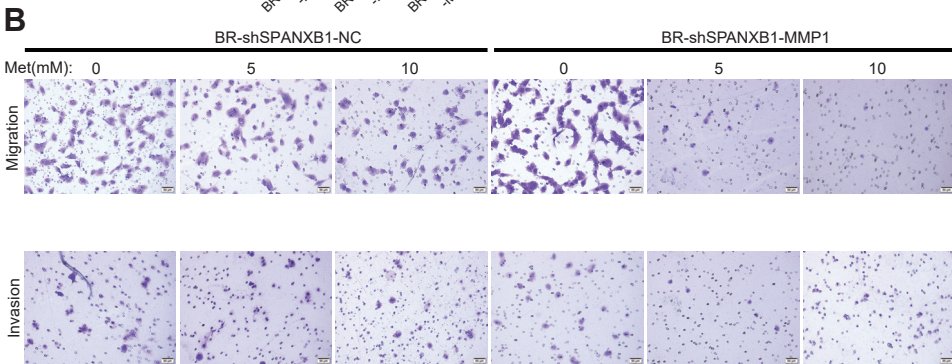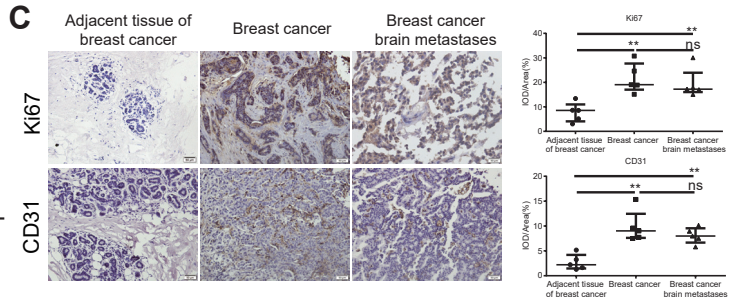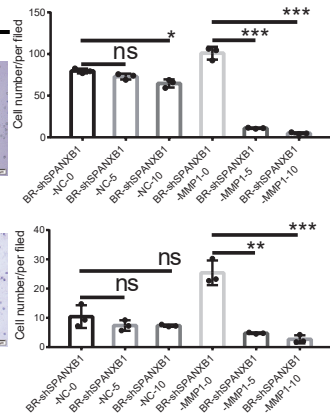

Supplement: Supplementary file 6 — Figure s5 [file 41420_2025_2721_MOESM6_ESM.pdf]

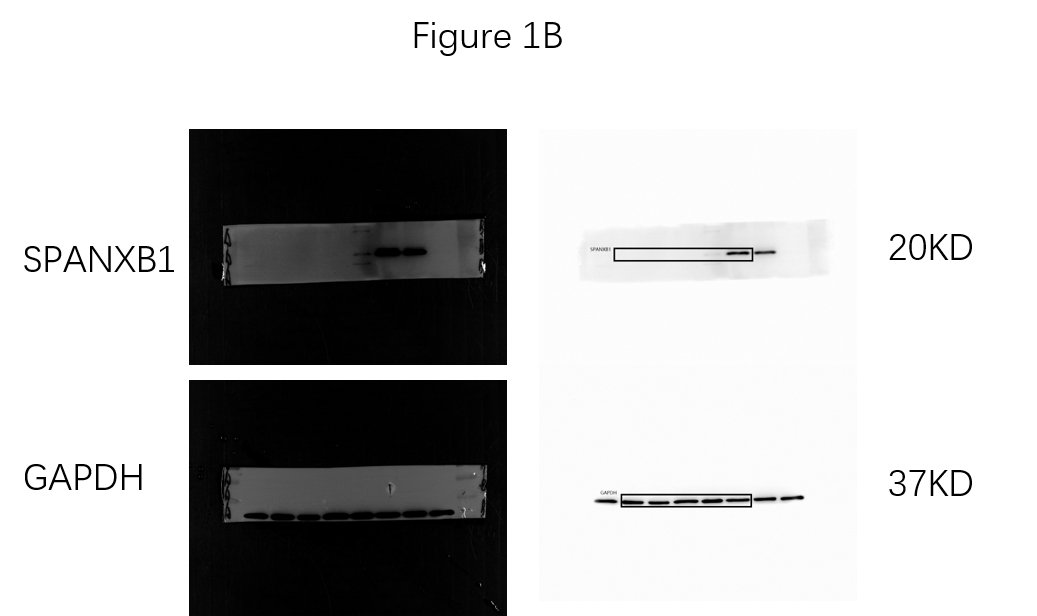

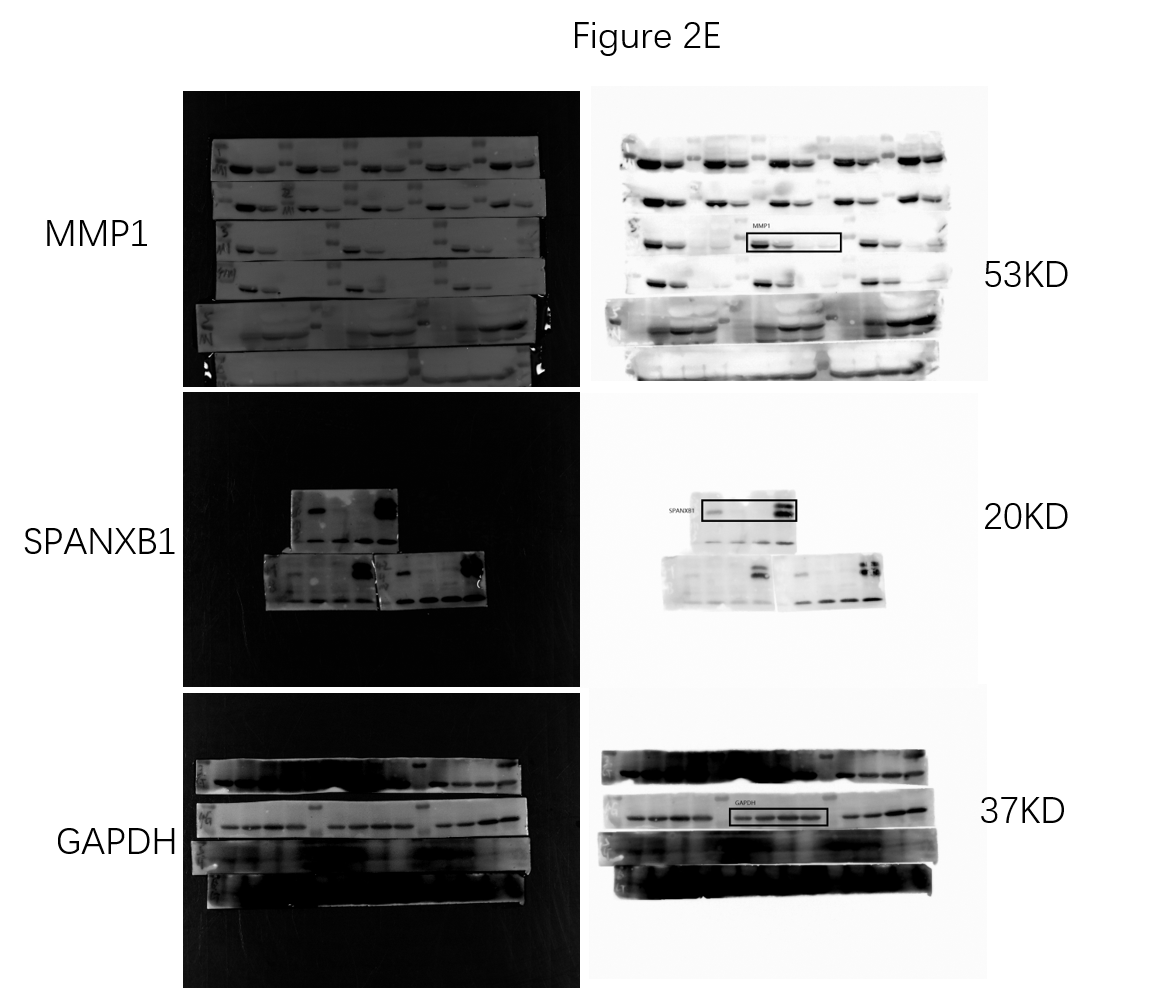


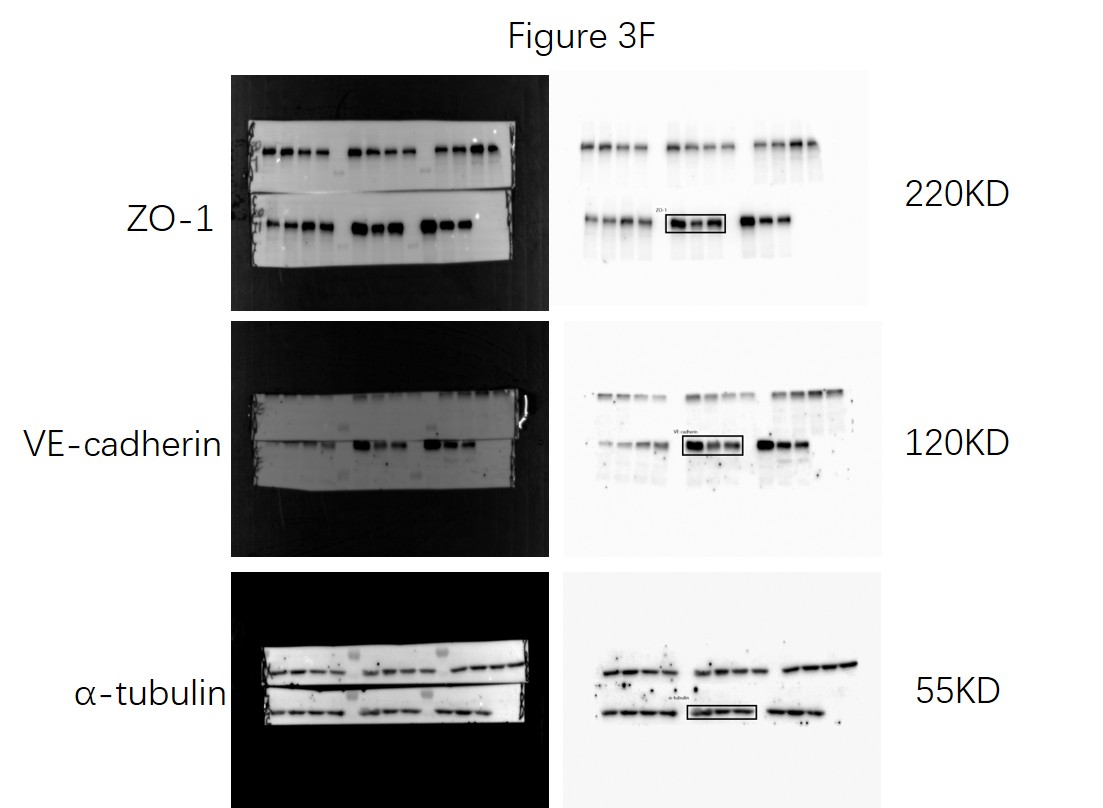

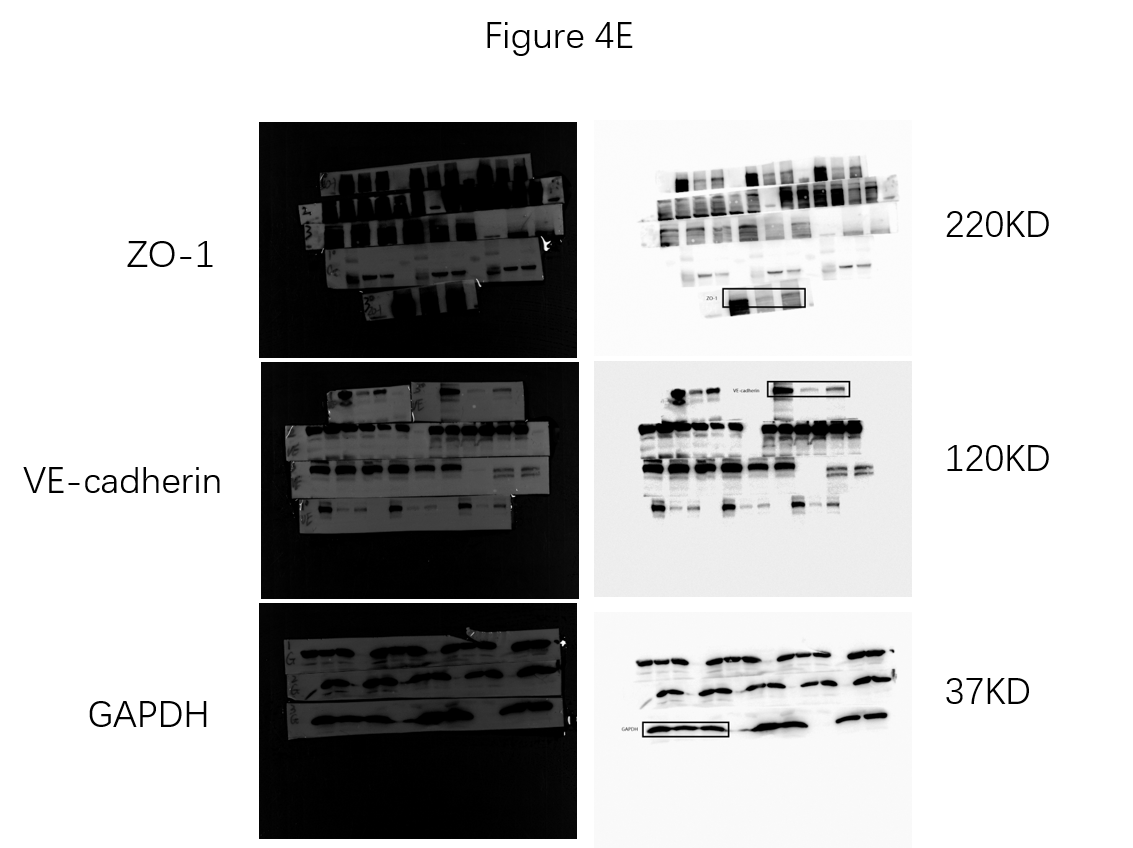


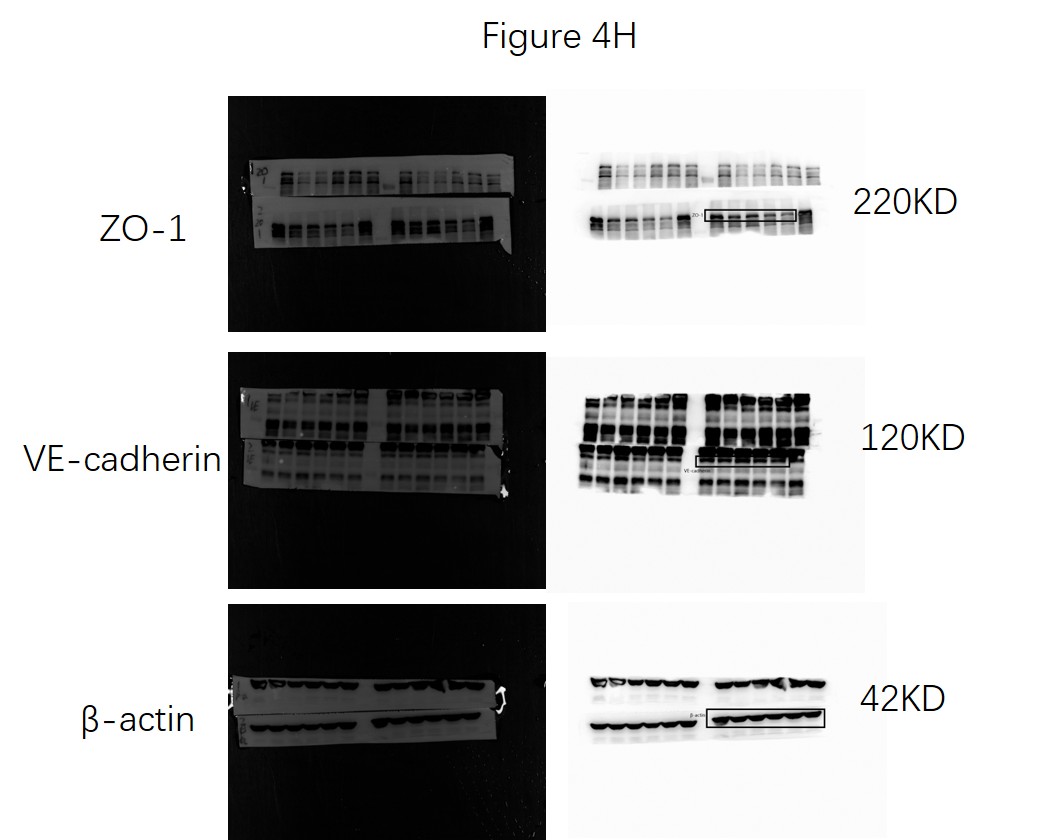

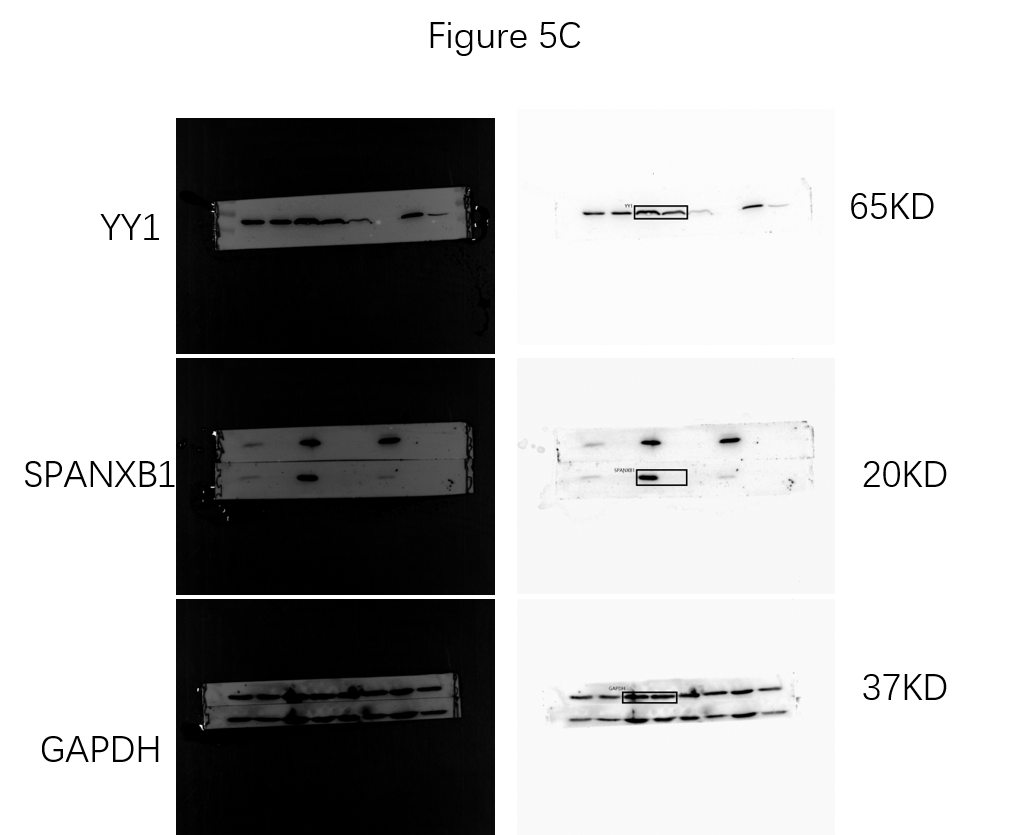

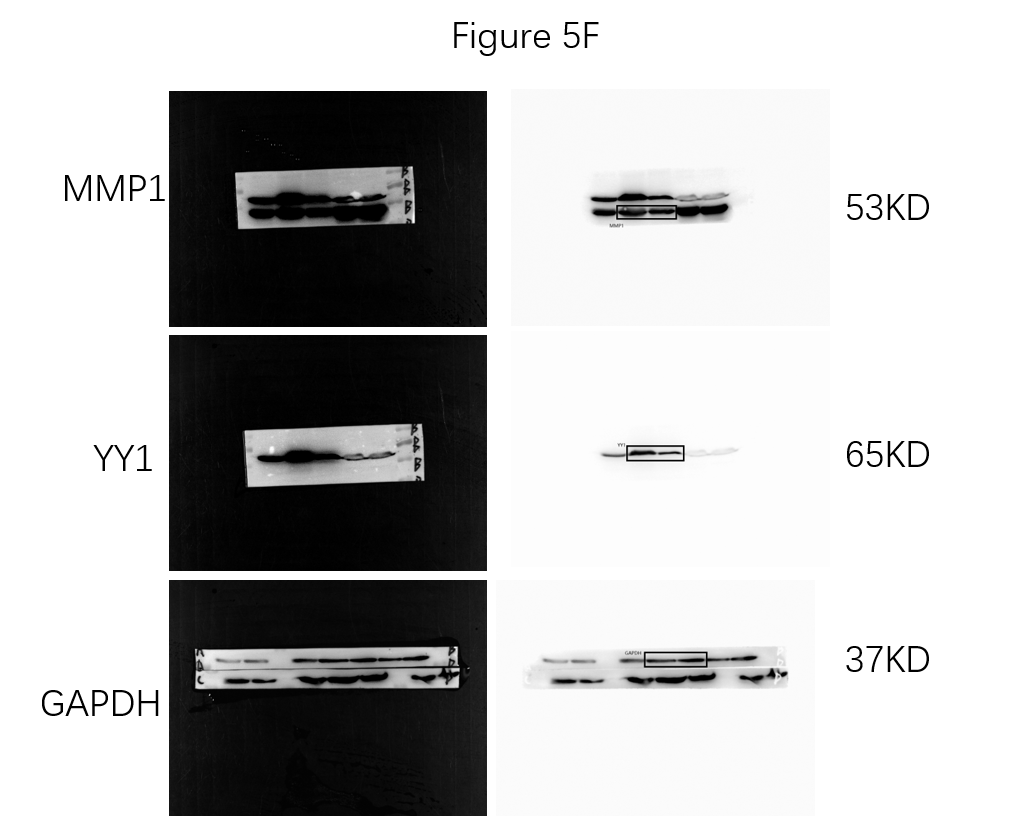

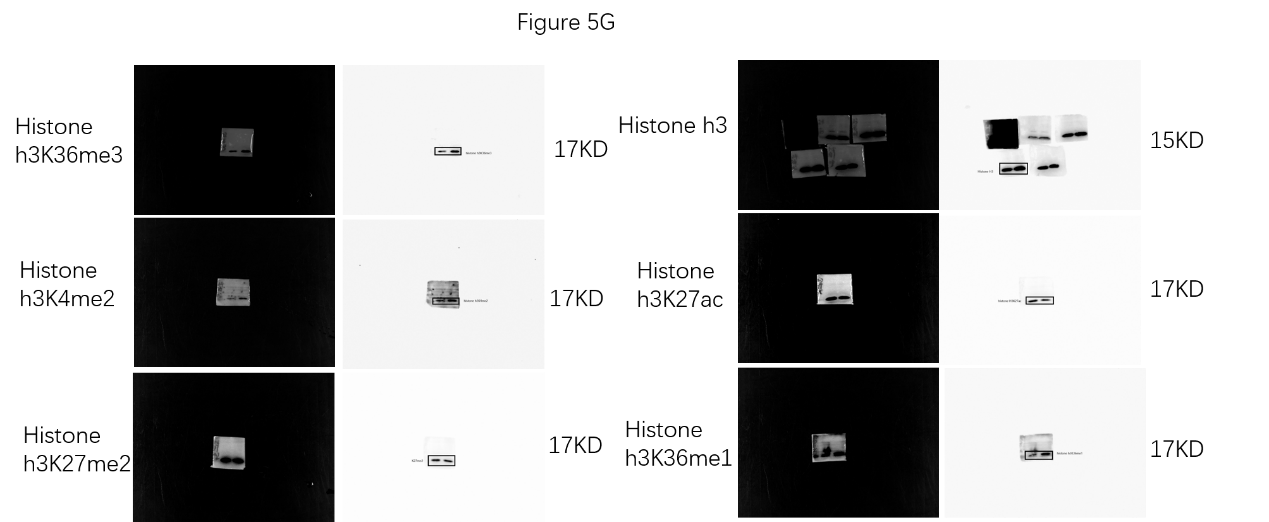


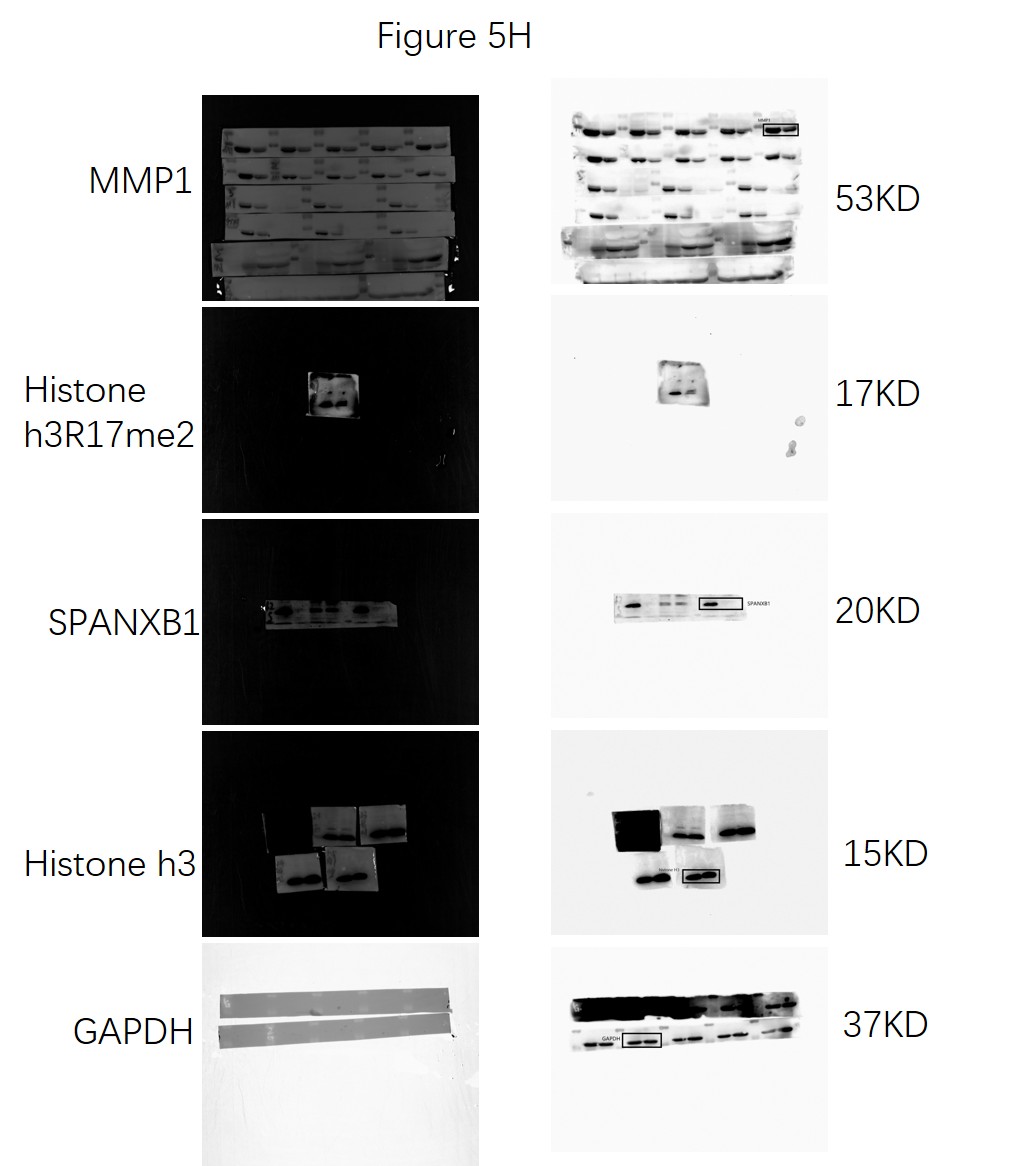

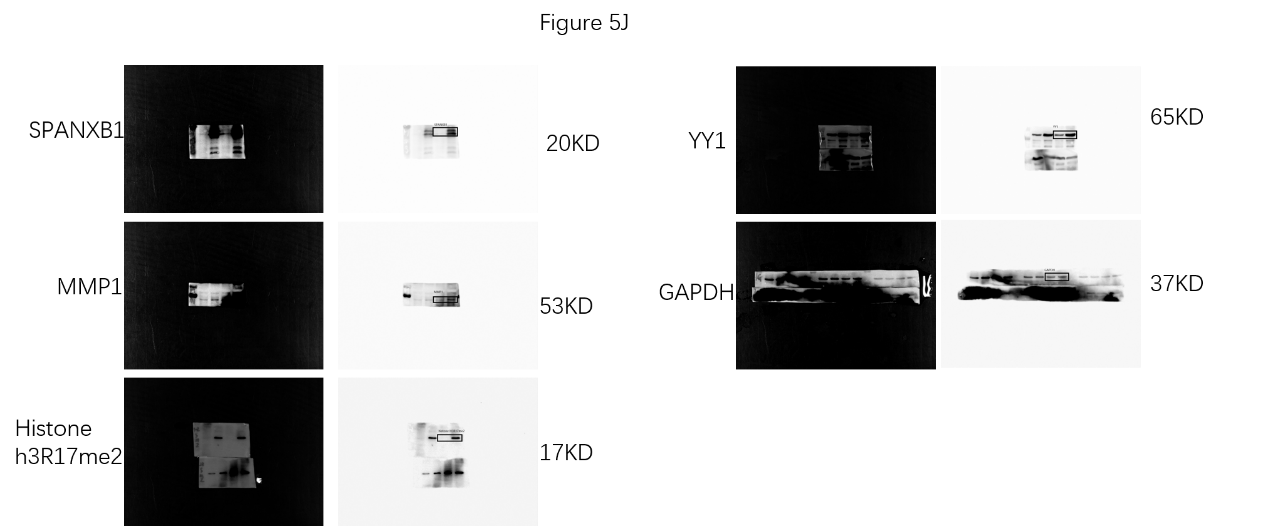

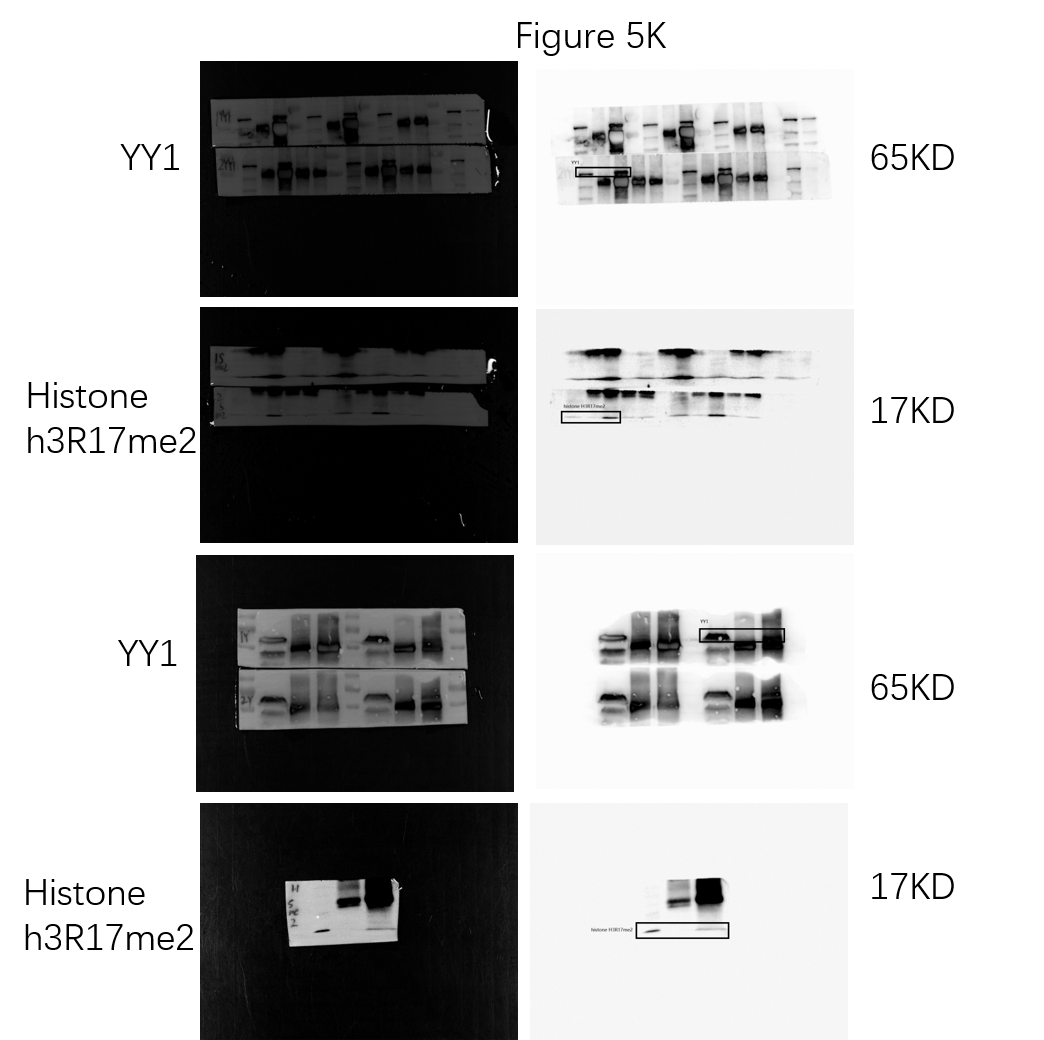

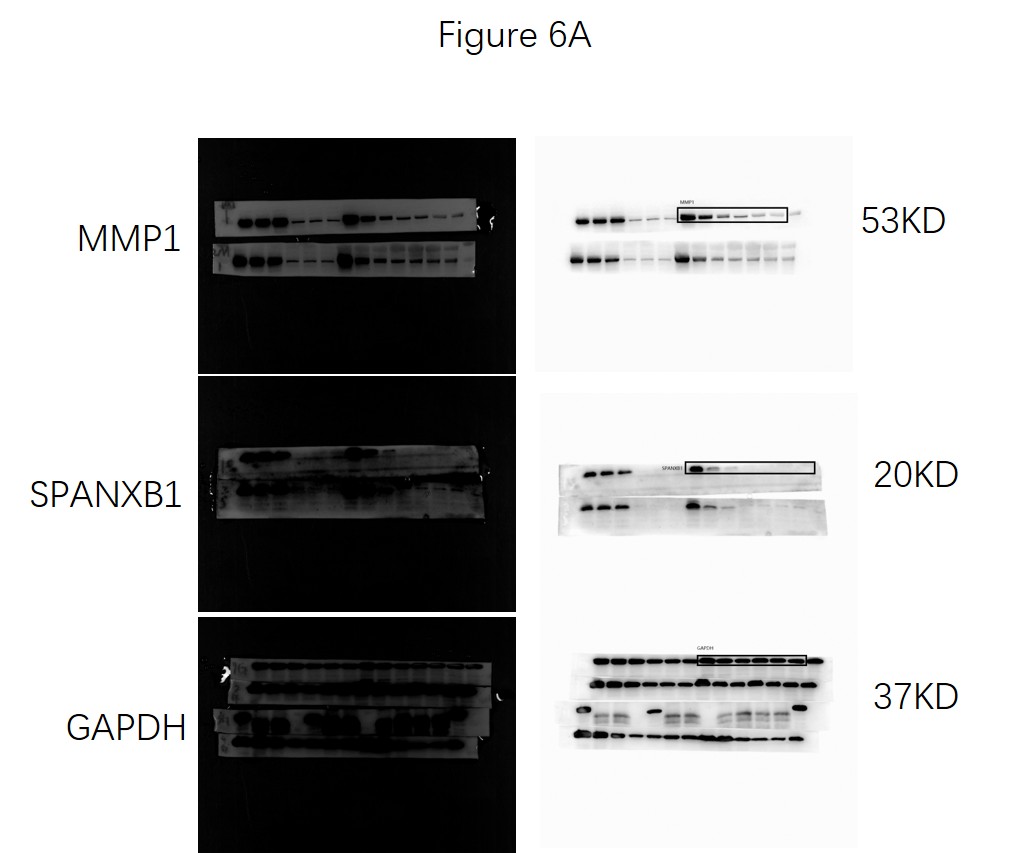

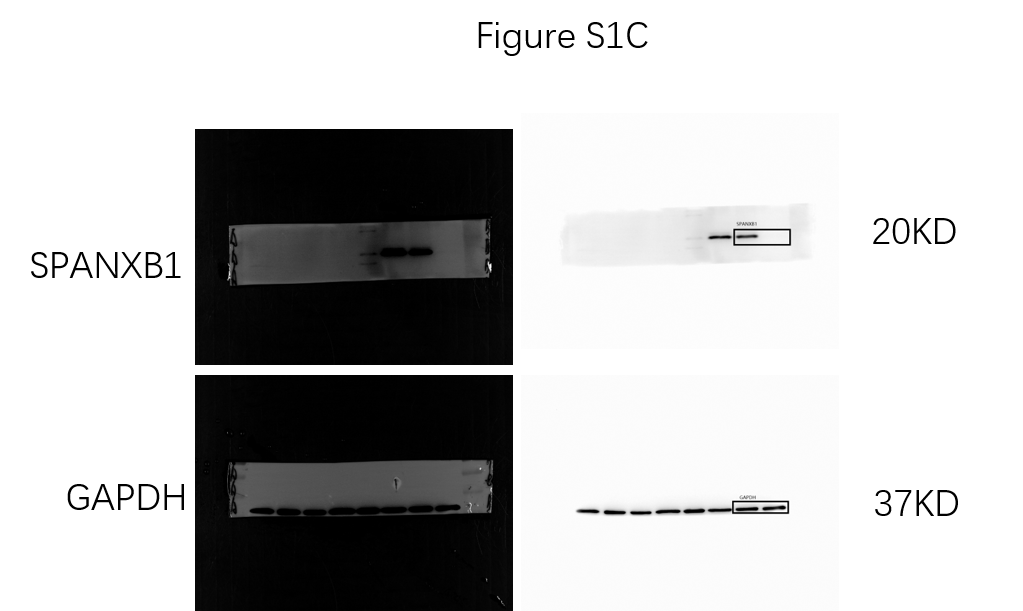

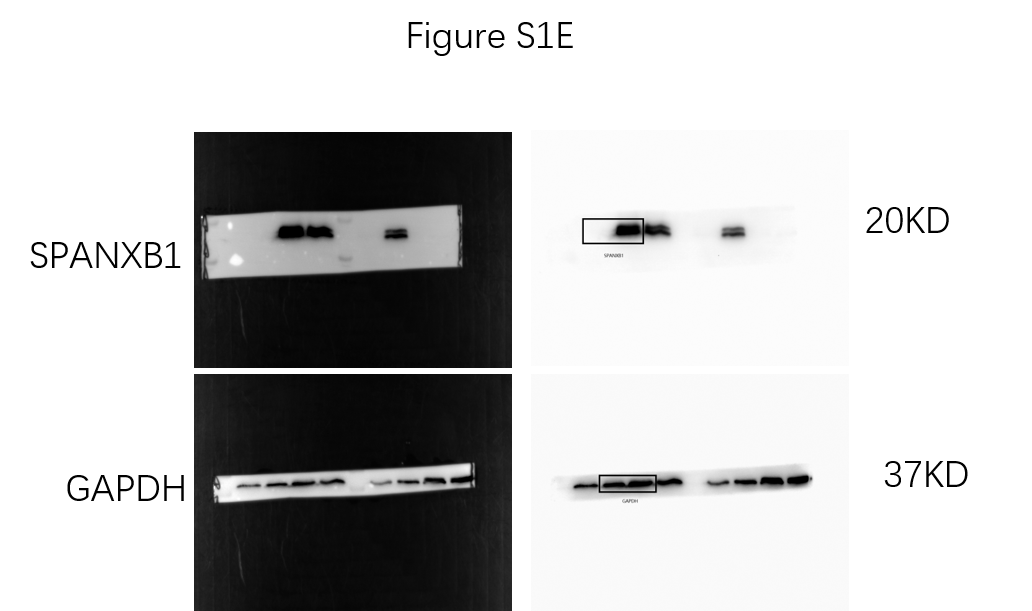

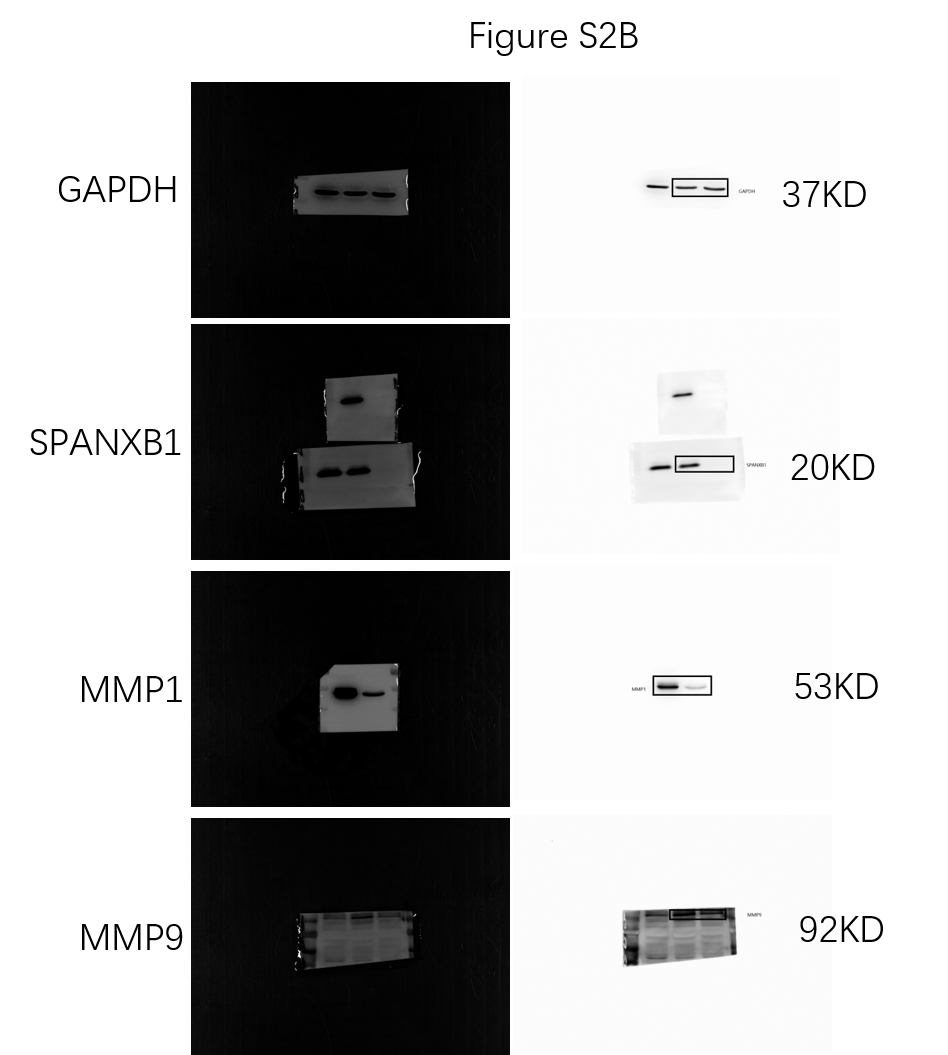

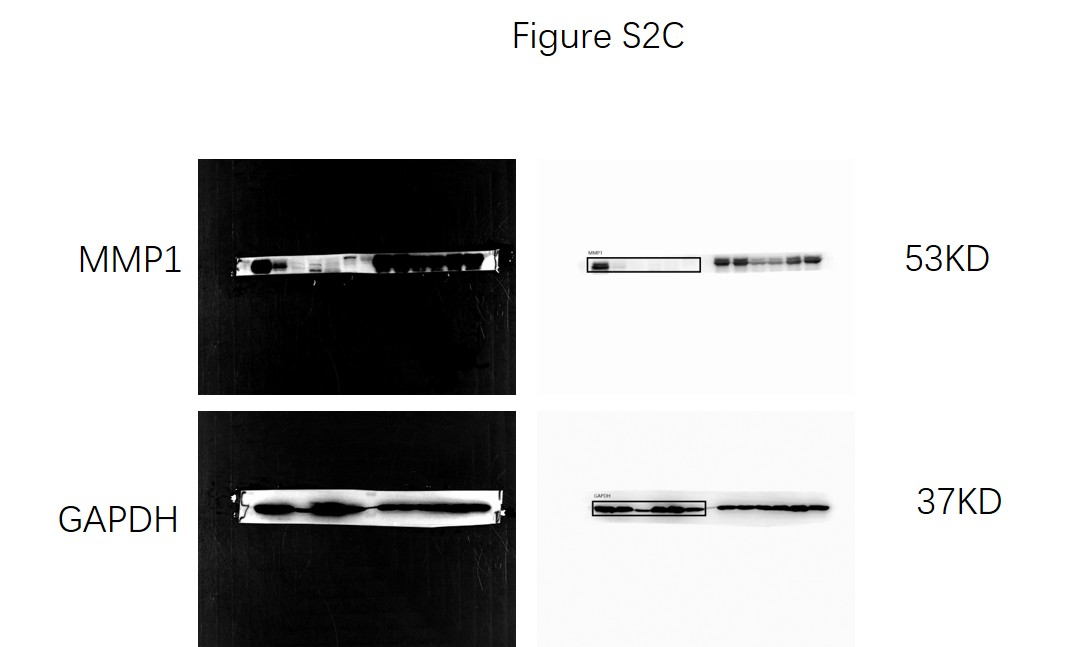

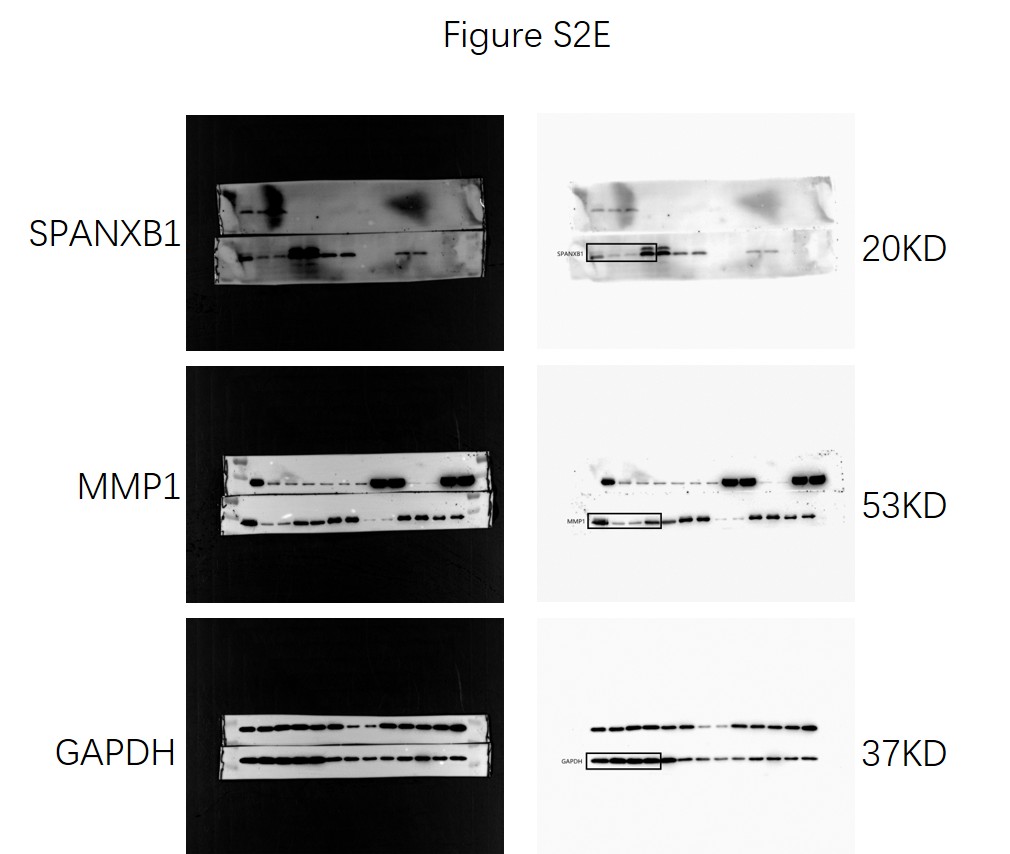

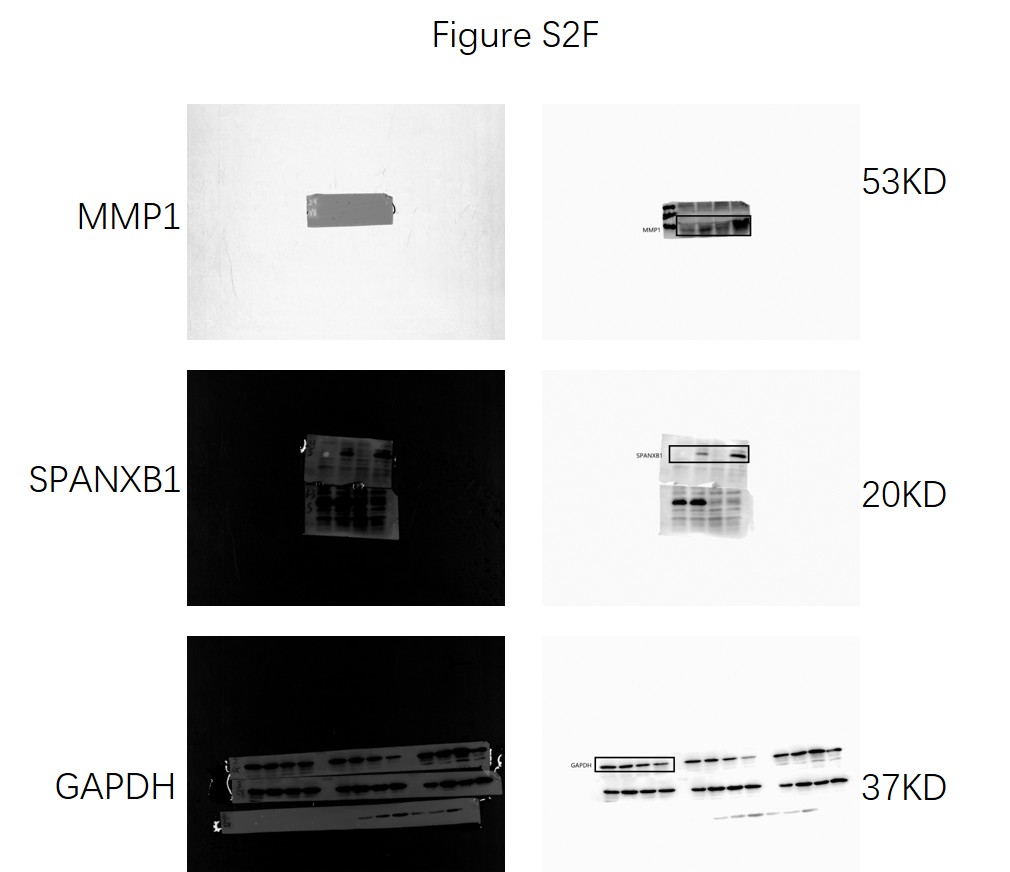


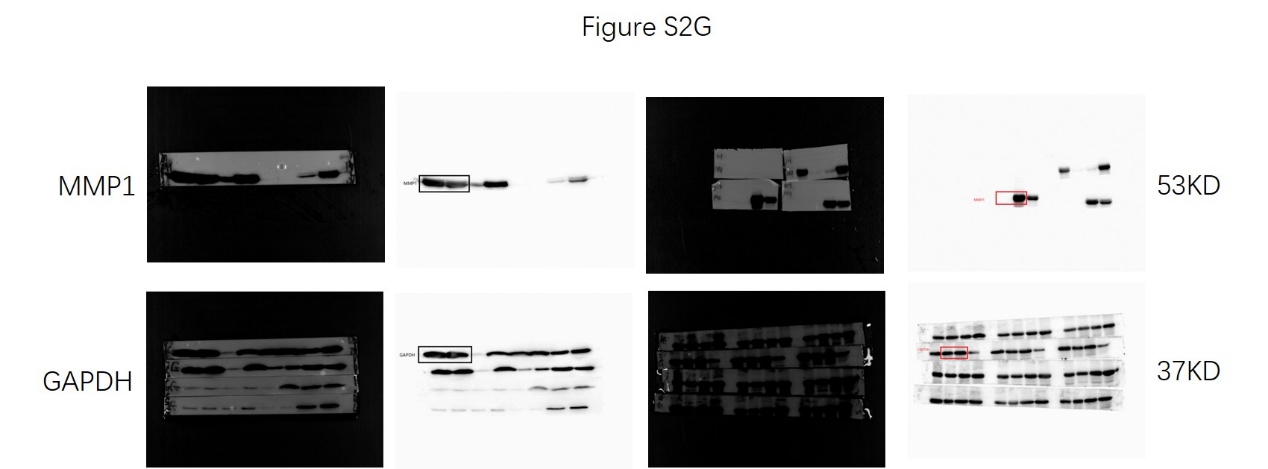

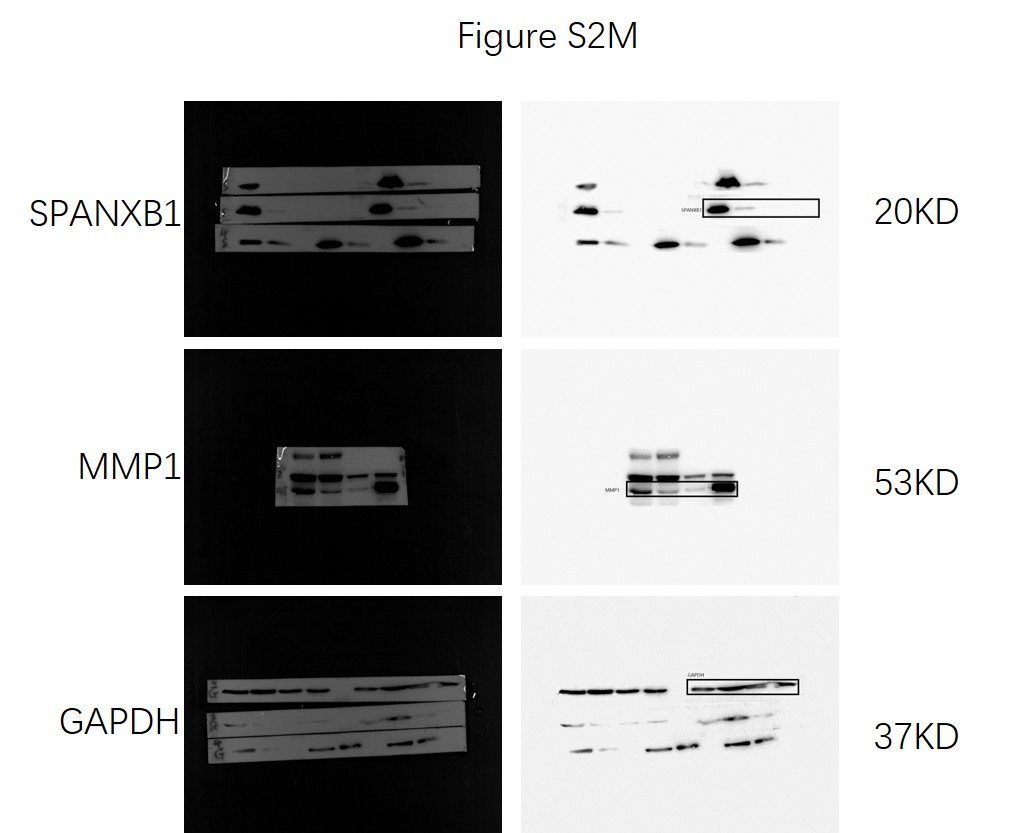

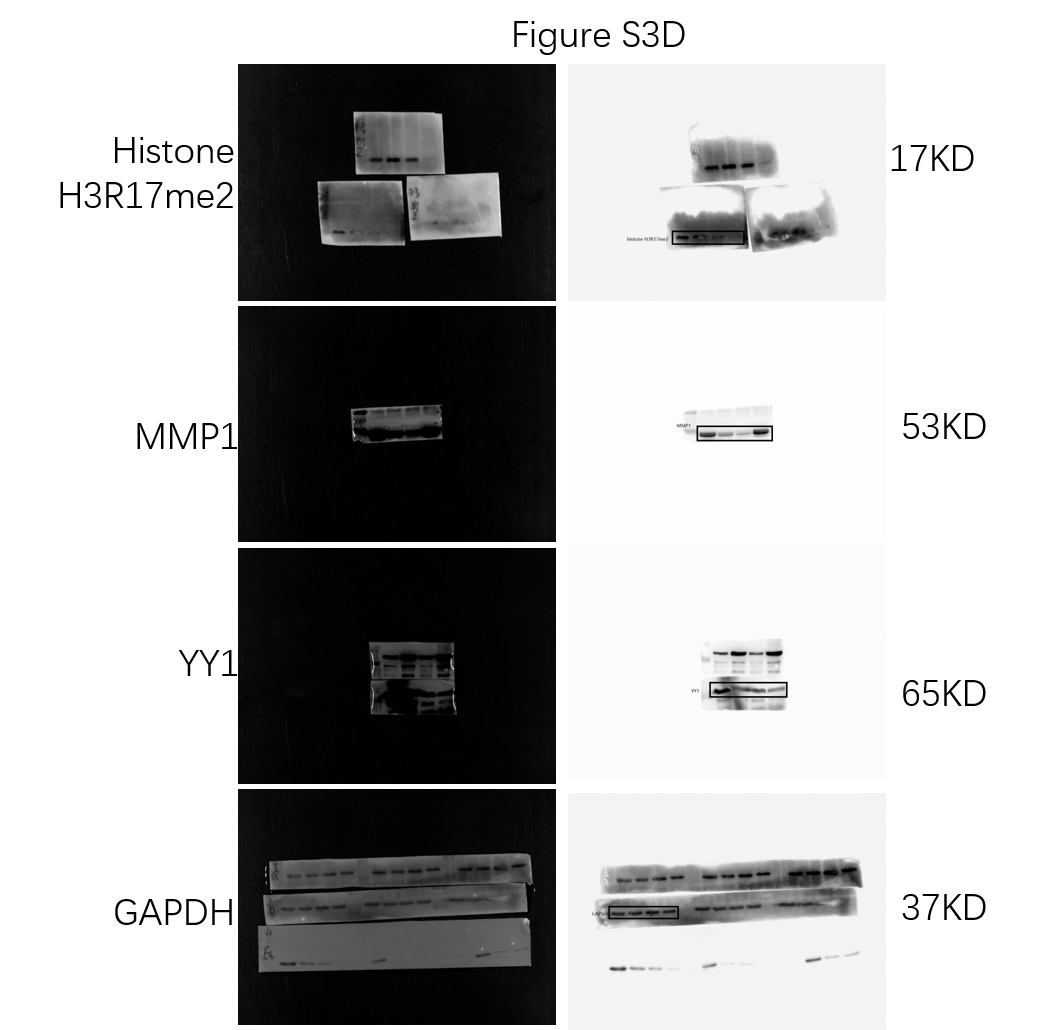

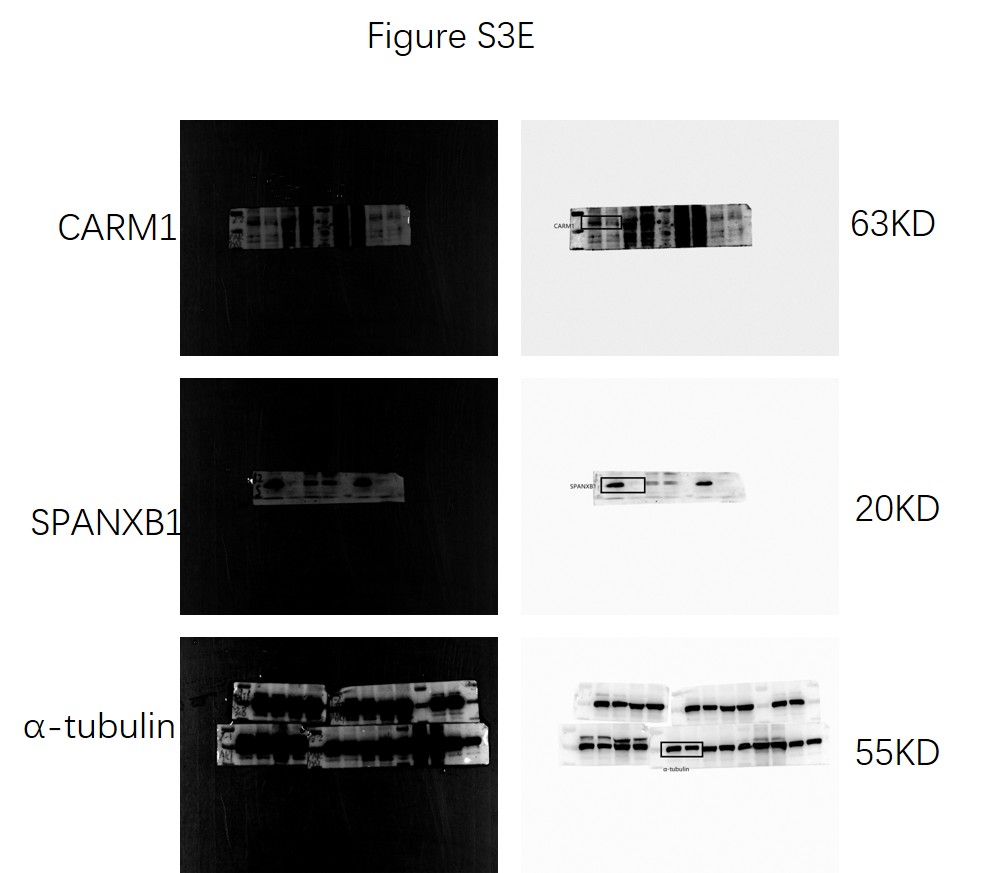

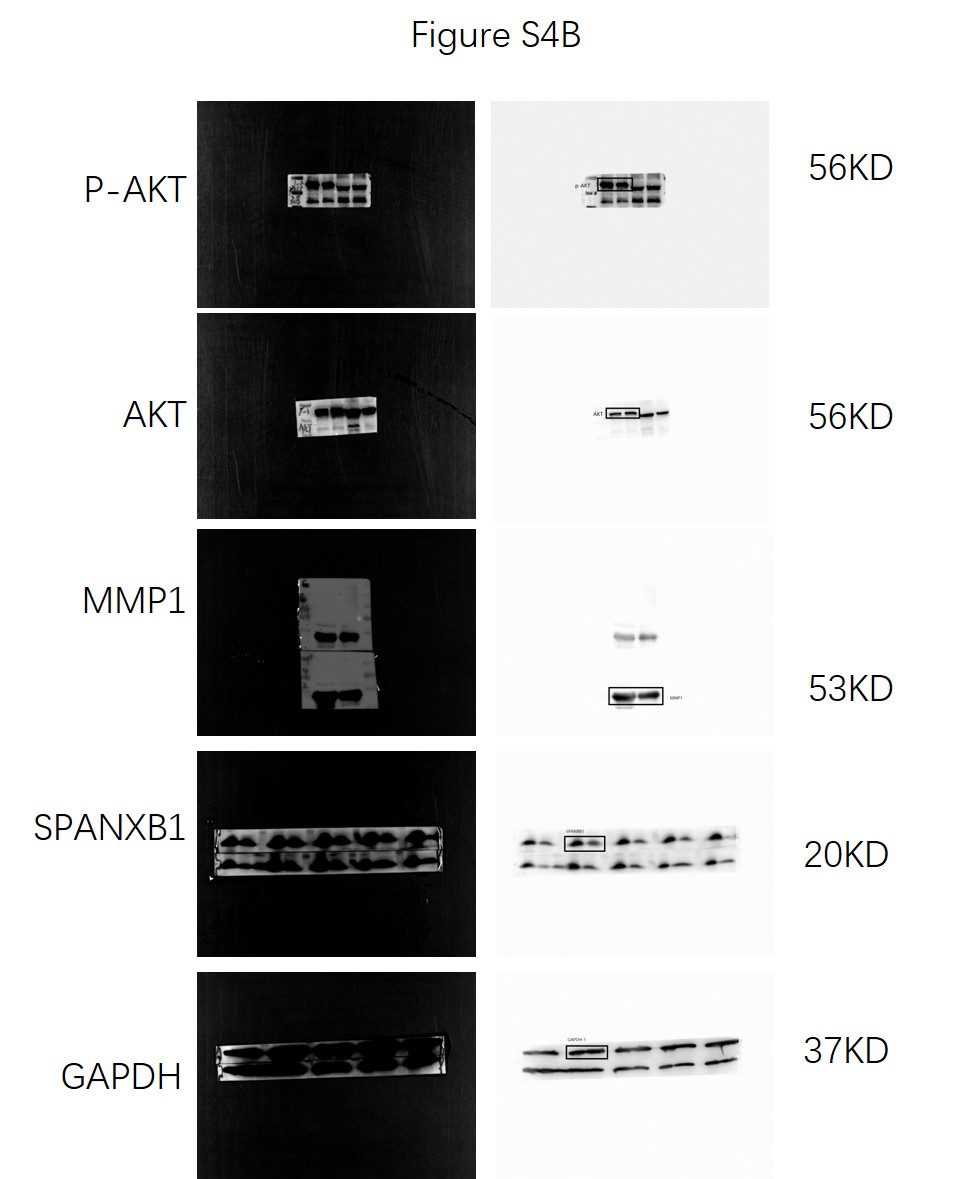

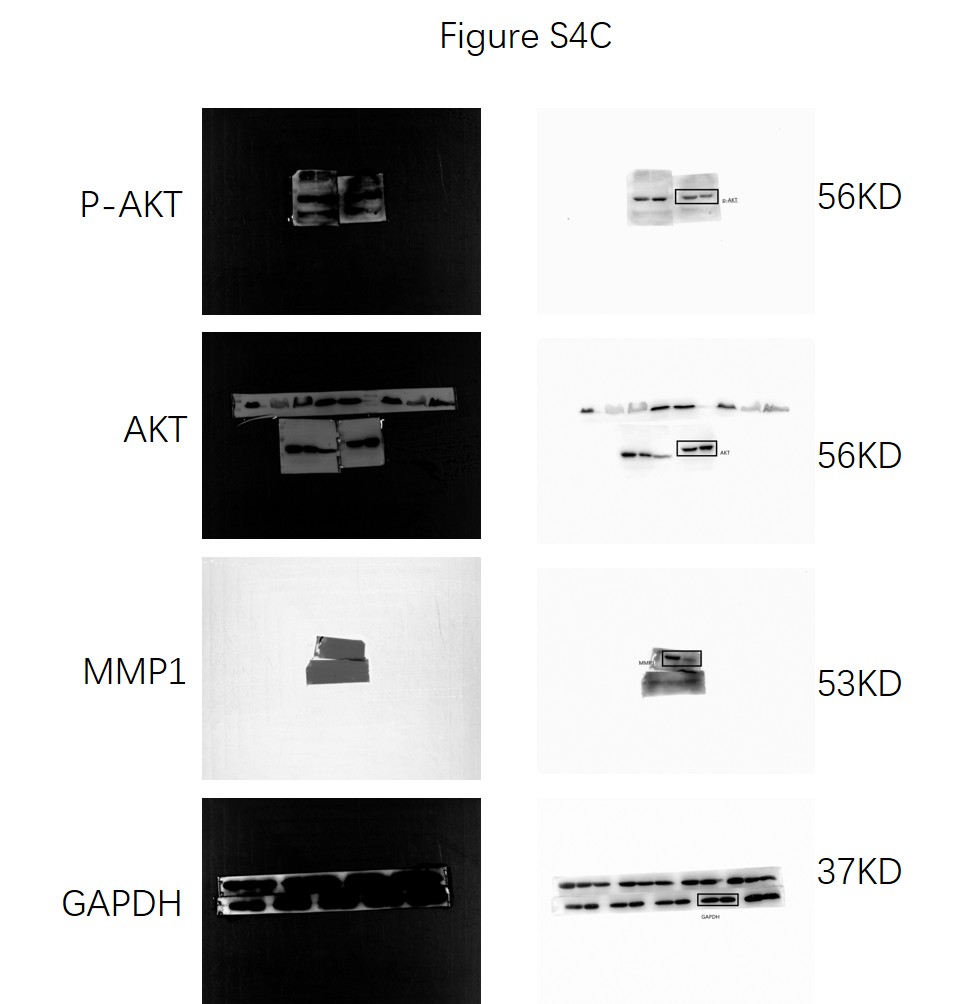

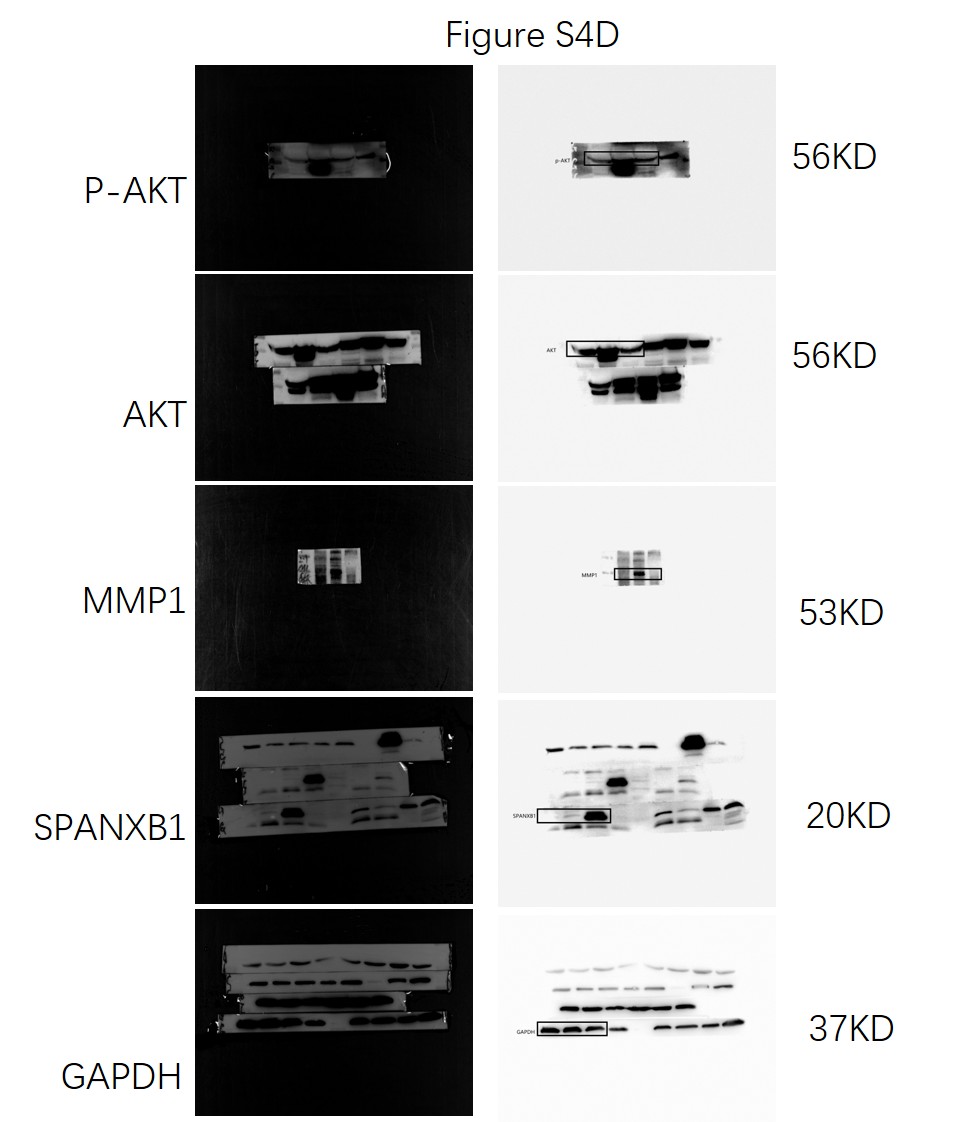

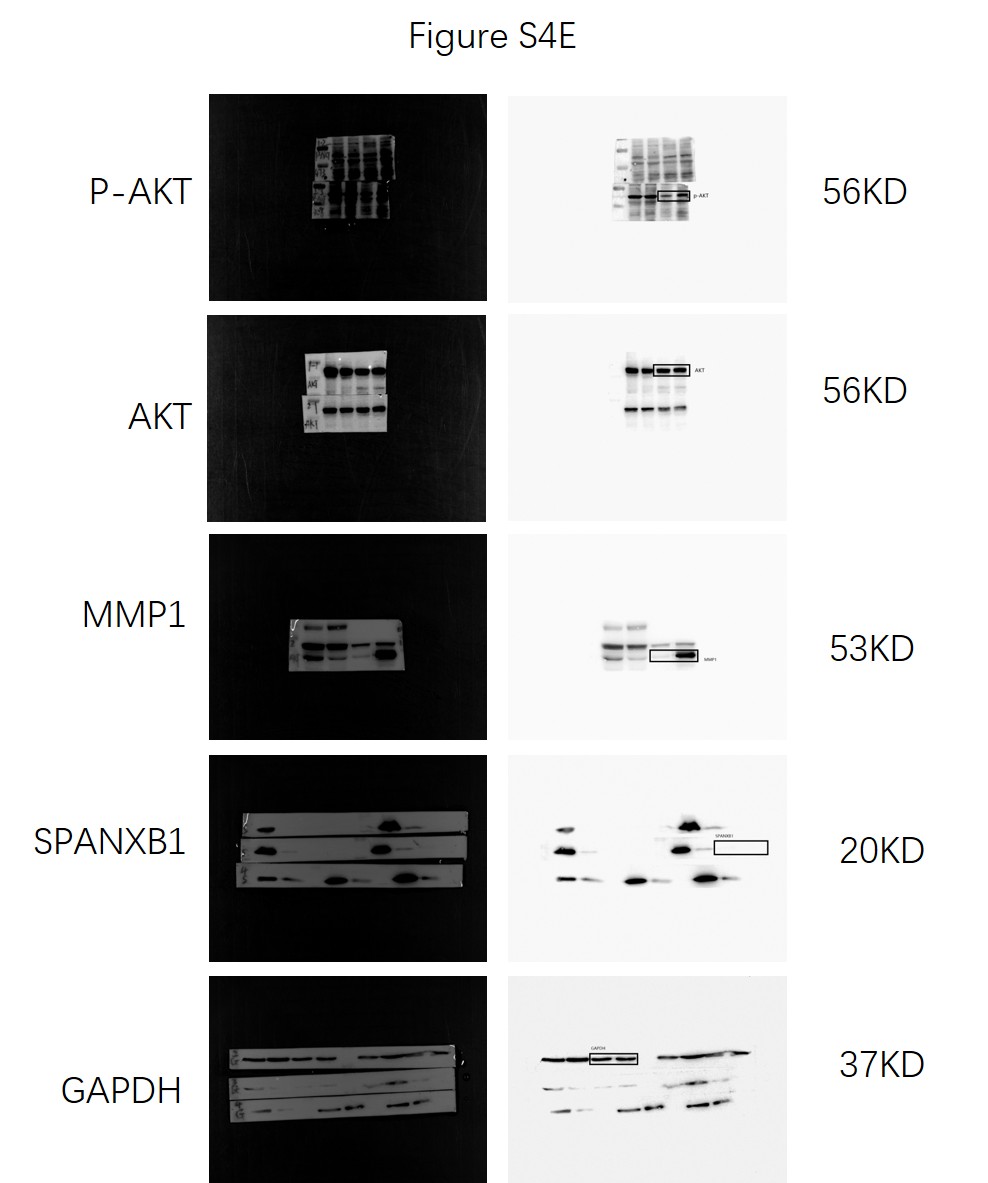

Supplement: Supplementary file 8 — Original Western Blots [file 41420_2025_2721_MOESM8_ESM.docx]
