# Supplementary material for: SPANXB1 drives brain metastasis in breast cancer via MMP1 regulation: potential therapeutic insights with metformin
Source: Cell Death Discov. 2025 Aug 30;11:418. doi: 10.1038/s41420-025-02721-4 (PMC12398519; doi:10.1038/s41420-025-02721-4)
Supplement: Supplementary file 7 — Table S1-4 [file 41420_2025_2721_MOESM7_ESM.docx]

Table S1: Sequences of siRNA targeting SPANXB1

| **gene** | **5'-3'** |
| --- | --- |
| siSPANXB1-1-sense | AGGCCAAUGAGGCCAACAAGA |
| siSPANXB1-1-antisense | UCUUGUUGGCCUCAUUGGCCU |
| siSPANXB1-2-sense | CGAGGCCAAUGAGGCCAACAA |
| siSPANXB1-2-antisense | UUGUUGGCCUCAUUGGCCUCG |
| siSPANXB1-3-sense | GAGGAGGAGGAAUUCAUAGAA |
| siSPANXB1-3-antisense | UUCUAUGAAUUCCUCCUCCUC |

Table S2: Sequences of shRNA for knockdown

| **gene** | **5'-3'** |
| --- | --- |
| shMMP1 | GCCTTCCAACTCTGGTAAT |
| shYY1 | CGATGGTTGTAATAAGAAGTT |
| shSPANXB1 | GAGGAGGAGGAATTCATAGAA |

Table S3: Primer sequences for qPCR analysis

| **gene name** | **5’-3’** |
| --- | --- |
| MMP2 | Forward Primer: AATACCATCGAGACCATGC  Reverse Primer: GTCCAGATCAGGTGTGTAGC |
| VIMENTIN | Forward Primer: GACGCCATCAACACCGAGTT  Reverse Primer: CTTTGTCGTTGGTTAGCTGGT |
| MMP1 | Forward Primer: GCTAACCTTTGATGCTATAACTACGA  Reverse Primer: TTTGTGCGCATGTAGAATCTG |
| MMP9 | Forward Primer: CCTTTGGACACGCACG  Reverse Primer: CCTAGTCCTCAGGGCACT |
| FAK | Forward Primer: GCTCCCTTGCATCTTCCAGT  Reverse Primer: ATTGCAGCCCTTGTCCGTTA |
| SPANXB1 | Forward Primer: CCAACAATCCAGTGTCCGCA  Reverse Primer: GTTCCTCCTGTAGCGAACCA |
| YY1 | Forward Primer: ACGGCTTCGAGGATCAGATTC  Reverse Primer: TGACCAGCGTTTGTTCAATGT |
| GAPDH | Forward Primer: TCCAAAATCAAGTGGGGCGA  Reverse Primer: TGATGACCCTTTTGGCTCCC |

Table S4 Primer sequences for ChIP-qPCR targeting MMP1 gene

| **ChIP-qPCR** | **5’-3’** |
| --- | --- |
| MMP1-1 | Forward Primer: CAGGTGGTGTTTGGTTAC  Reverse Primer: ACTCAGGAGGAAAGGGTG |
| MMP1-2 | Forward Primer: CCCACCAGCAGTGTAGAAG  Reverse Primer: CAAGAATGCCCATAATCAA |
